# Histone H3 lysine 4 methyltransferase is required for facultative heterochromatin at specific loci

**DOI:** 10.1186/s12864-019-5729-7

**Published:** 2019-05-08

**Authors:** Qiaoqiao Zhu, Mukund Ramakrishnan, Jinhee Park, William J. Belden

**Affiliations:** 1 Department of Animal Sciences, Rutgers, The State University of New Jersey, New Brunswick, NJ 08901 USA; 2 Current Address: Department of Biological Sciences, IISER Berhampur, Berhampur, Ganjam, Odisha 760010 India

**Keywords:** Histone lysine methyltransferase, Heterochromatin, Histone H3 lysine 4 methylation, Histone H3 lysine 9 methylation, Long non-coding RNA, Light-activated gene expression

## Abstract

**Background:**

Histone H3 lysine 4 tri-methylation (H3K4me3) and histone H3 lysine 9 tri-methylation (H3K9me3) are widely perceived to be opposing and often mutually exclusive chromatin modifications. However, both are needed for certain light-activated genes in *Neurospora crassa* (Neurospora), including *frequency (frq)* and *vivid* (*vvd*). Except for these 2 loci, little is known about how H3K4me3 and H3K9me3 impact and contribute to light-regulated gene expression.

**Results:**

In this report, we performed a multi-dimensional genomic analysis to understand the role of H3K4me3 and H3K9me3 using the Neurospora light response as the system. RNA-seq on strains lacking H3 lysine 4 methyltransferase (KMT2/SET-1) and histone H3 lysine 9 methyltransferase (KMT1/DIM-5) revealed some light-activated genes had altered expression, but the light response was largely intact. Comparing these 2 mutants to wild-type (WT), we found that roughly equal numbers of genes showed elevated and reduced expression in the dark and the light making the environmental stimulus somewhat ancillary to the genome-wide effects. ChIP-seq experiments revealed H3K4me3 and H3K9me3 had only minor changes in response to light in WT, but there were notable alterations in H3K4me3 in *Δkmt1/Δdim-5* and H3K9me3 in *Δkmt2/Δset-1* indicating crosstalk and redistribution between the modifications. Integrated analysis of the RNA-seq and ChIP-seq highlighted context-dependent roles for KMT2/SET1 and KMT1/DIM-5 as either co-activators or co-repressors with some overlap as co-regulators. At a small subset of loci, H3K4 methylation is required for H3K9me3-mediated facultative heterochromatin including, the central clock gene *frequency* (*frq*). Finally, we used sequential ChIP (re-ChIP) experiment to confirm Neurospora contains K4/K9 bivalent domains.

**Conclusions:**

Collectively, these data indicate there are obfuscated regulatory roles for H3K4 methylation and H3K9 methylation depending on genome location with some minor overlap and co-dependency.

**Electronic supplementary material:**

The online version of this article (10.1186/s12864-019-5729-7) contains supplementary material, which is available to authorized users.

## Background

The Neurospora light response is a transcriptional cascade that responds to environmental cues and controls circadian entrainment and developmental programs [1–4]. White Collar-1 (WC-1) and WC-2 are GATA-type transcription factors that drive light-mediated expression and associate via PAS (Per, Arnt and SIM) domains to form the White collar complex (WCC) [5–7]. WC-1 serves as the photoreceptor sensing blue light via its LOV (light, oxygen and voltage) domain in a photochemical reaction involving a cysteinyl-flavin adenine dinucleotide (FAD) adduct [8, 9]. Microarray and RNA-seq experiments indicate that approximately 3–20% of all genes change expression in response to light and fall into 1 or 3 categories; early light-responsive that require the WCC, late light-responsive that require SUB-1, and light-repressed [10–13]. There are also light-activated changes to chromatin structure and nucleosome position mediated by ATP-dependent chromatin-remodeling enzymes [12, 14, 15]. The nucleosome movement and remodeling is likely initiated by the Neurospora homologue of GCN5 (NGF-1), which acetylates histone H3 on lysine 14 and is needed for proper light-activated gene expression [16]. Despite our knowledge of WCC, SUB-1, NGF-1, chromatin remodeling and RNA polymerase II (PolII) activity, the role of histone modifications in the Neurospora light response is still very limited.

Previously, we demonstrated that KMT2/SET-1-dependent histone H3 lysine 4 methylation and KMT1/DIM-5-dependent histone H3 lysine 9 methylation are involved in light- and clock-regulated gene expression playing a supporting role in photoadaptation and negative feedback inhibition [17, 18]. Histone H3 lysine 4 tri-methylation (H3K4me3) is generally viewed as a modification supporting transcriptional activation because it is enriched in active genes [19] even though H3K4me3 was originally found to be involved in repression [20–22]. The role of KMT2/SET-1 in repression is not fully understood, but studies in *S. pombe* indicate it is needed for repression at subtelomeric genes and retrotransposons, possibly independent of H3K4 methylation [23–25]. Moreover, recent models suggest that H3K4me3 may be less of mark for activation, and more reflective of cell-specific transcriptional states [26, 27]. Thus, even though KMT2/SET-1-dependent H3K4 methylation is generally associated with euchromatin, KMT2/SET-1 appears to have a context dependent role in silencing. In contrast, H3K9me3, which is typically bound by Heterochromatin Protein 1 (HP1), is entrenched as a repressive modification underlying condensed heterochromatin [28–30]. Yet evidence suggests H3K9me3 can also be found in actively transcribed genes in mammals [31] and heterochromatic regions (e.g. centromeres and telomeres) are often transcriptionally active due to the requirement of RNAi-mediated heterochromatin [32]. In Neurospora, it is widely believed there is little, if any cross talk between KMT2/SET-1 and KMT1/DIM-5 because H3K9me3 and H3K4me2 appear to be mutually exclusive [33] and Neurospora centromeres lack H3K4 methylation [34]. However, both KMT2/SET-1 and KMT1/DIM-5 assist circadian negative feedback of the clock *frequency* (*frq*), and both are required for DNA methylation (5^m^C) at *frq.* In addition, the deletion strains appear to have some effect on photoadaptation and H3K4me3 and H3K9me3 appear to peak within *frq* approximately 30 min after exposure to light [17, 18], a time when VIVID (VVD)-mediated down-regulation and adaptation has commenced [35, 36]. Specifically, the loss of DNA methylation at *frq* in *Δkmt2/Δset-1* implicates H3K4me3 in KMT2/DIM-5-dependent facultative heterochromatin and counters prevailing views on H3K4 methylation.

The dynamic DNA methylation and facultative heterochromatin at *frq* also require coordinated expression of a light-activated long non-coding natural antisense transcript (NAT) *qrf* [37–39]. Strains with constitutive low-level expression of *qrf* have a localized defect in DNA methylation and heterochromatin [37, 40]. As an aside, the initial burst of light-activated *qrf* appears to contribute to bimodal regulation of *frq* by initially creating a more permissive state for expression prior to heterochromatin-mediated silencing [37]. The role of *qrf* in establishing heterochromatin may not be unique because many sense-antisense pairs and convergent transcripts give rise to Dicer-independent small interfering RNA (disiRNA) and subsequent DNA methylation [39, 41].

In order to further understand the role of KMT2/SET-1, KMT1/DIM-5 and long noncoding RNA (lncRNA), we took a genomics approach and examined the connections between H3K4me3 and H3K9me3 and how these impact gene expression in the Neurospora light response. Part of the premise is that, at least at *frq,* KMT2/SET-1 and KMT1/DIM-5 along with an antisense transcript are all needed for heterochromatin. Therefore, we coupled RNA-seq and transcript discovery with H3K4me3 and H3K9me ChIP-seq. Our results reveal conventional paradigms surrounding H3K4me3 and H3K9me3 are not entirely universal and point to a context, inter-dependent nature for both modifications. We have found that at some loci, KMT2/SET1 and KMT1/DIM-5 are involved in repression, and at other loci, they aid in co-activation. In addition, KMT1/DIM-5 and KMT2/SET-1 can function alone or in combination to help modulate gene regulation. The data reveal that H3K4me3 and H3K9me3 are not solely confined to activation or repression and instead point to a complex combinatorial relationship between these modifications and highlight the need for more mechanistic studies.

## Results

### Role of KMT1/DIM-5 and KMT2/SET-1 in transcriptional regulation

**Fig. 1:**
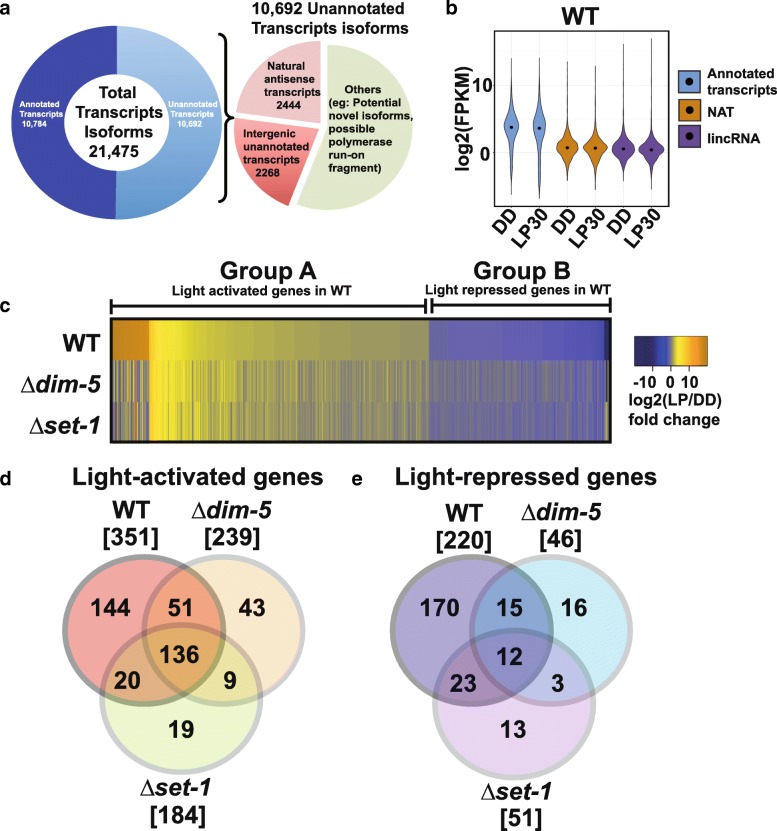
RNA-seq analysis in WT, *∆kmt1/∆dim-5* and *∆kmt2/∆set-1*. (**a**) Graphical representation of identified transcripts. In total, there were 21,475 unique transcripts classified as 10,784 previously annotated protein-coding transcripts and 10,692 unannotated transcripts, that were further subdivided into 2444 natural antisense transcripts and 2268 predicted lincRNAs. (**b**) Violin plot depicting the expression levels (FPKM, log2) of transcripts belonging to annotated transcripts, NATs and lincRNAs. The black dots indicate median. (**c**) Heatmap showing the clustering of light-activated and light-repressed genes in WT (q < 0.05) and compared to the same genes in *∆kmt1/∆dim-5* and *∆kmt2/∆set-1*. Group A includes genes that were light-activated in WT while Group B represents genes that were light-repressed. Relative expression levels are shown as log2 fold change of LP vs. DD as indicated in the range bar. The overlap among (**d**) light-activated (q < 0.05) or (**e**) light-repressed (q < 0.05) genes are represented as Venn diagrams

In order to understand the role of KMT1 and KMT2 in the Neurospora light response, we performed a comprehensive RNA-sequencing (RNA-seq) on *Δkmt1/Δdim-5* and *Δkmt2/Δset-1* compared to WT with RNA isolated from mycelia grown in the dark for 24 h (DD24) and after a 30-min light exposure (LP30). The RNA-seq was performed on ribo-depleted RNA and we preserved the DNA strand during library preparation to identify lncRNAs, NATs and to monitor potential spurious transcription in the mutants. We used HISAT2 and StringTie for mapping and transcript identification [42] (Additional file 1a). HISAT2 and StringTie were chosen because other mapping and transcript discovery algorithms were unable to identify the *frq* NAT, *qrf,* and we felt this was a minimum requisite to ensure confidence in transcript discovery. In total, we identified 21,475 isoform level transcripts; 10,784 are annotated protein-coding genes and 10,692 were transcripts not present in the NC12 annotation. Further predictions indicated there are 2444 NATs and 2268 long intergenic noncoding RNAs (lincRNAs) (Fig. 1a) from the unannotated transcripts. The absolute number of lincRNAs and NATs is slightly larger, but consistent with previous reports and the small differences are likely due to different stringency constraints used during identification [43, 44]. We also performed multidimensional scaling (MDS) on the replicates to gage the variance among the strains and conditions and found the deletions contributed more to the changes in gene expression compared to environmental stimulus (Additional file 1b) Next, we examined the expression level distribution among the different transcript types in WT under DD and LP30 and found that NATs and lincRNA are typically expressed at a much lower level than annotated protein-coding genes (Fig. 1b). Similar results were found for *Δkmt1/Δdim-5* (Additional file 2a) and *Δkmt2/Δset-1* (Additional file 2b), and we did not detect major global differences among the strains (data not shown).

To examine the defects in transcription arising from loss of H3K4 and/or H3K9 methylation in the light, we compared the expression differences of *Δkmt1/Δdim-5* and *Δkmt2/Δset-1* to WT. Hierarchical clustering of light-regulated genes in each strain revealed many were not activated in *Δkmt1/Δdim-5* or *Δkmt2/Δset-1* while many light-repressed genes became activated (q < 0.05) (Fig. 1c, Additional file 3). Independent clustering of *Δkmt1/Δdim-5* and *Δkmt2/Δset-1* revealed analogous result where subsets of genes that became unresponsive while different sets became responsive (Additional file 3, Additional file 4a and b). Comparison of light-activated genes in WT, *Δkmt1/Δdim-5* and *Δkmt2/Δset-*1 revealed that 144 transcripts required both KMT1/DIM-5 and KMT2/SET-1, while 43 and 19 became light-activated in *Δkmt1/Δdim-5* and *Δkmt2/Δset-1,* respectively (Fig. 1d). In contrast, 170 of 220 light-repressed genes in WT required both KMT1/DIM-5 and KMT2/SET-1 (Fig. 1e). Among the light-activated genes in WT, 12.5% are predicted to be lincRNA and 3% are light-activated NATs (Additional file 4c). Collectively, the data indicate that although the light response is still largely intact, a subset of genes lose proper regulation in the absence of KMT1/DIM-5 or KMT2/SET-1 while other genes become light-activated or light-repressed in *Δkmt1/Δdim-5* or *Δkmt2/Δset-1*.

### KMT1/DIM-5 and KMT2/SET1 are co-activators and co-repressors

**Fig. 2:**
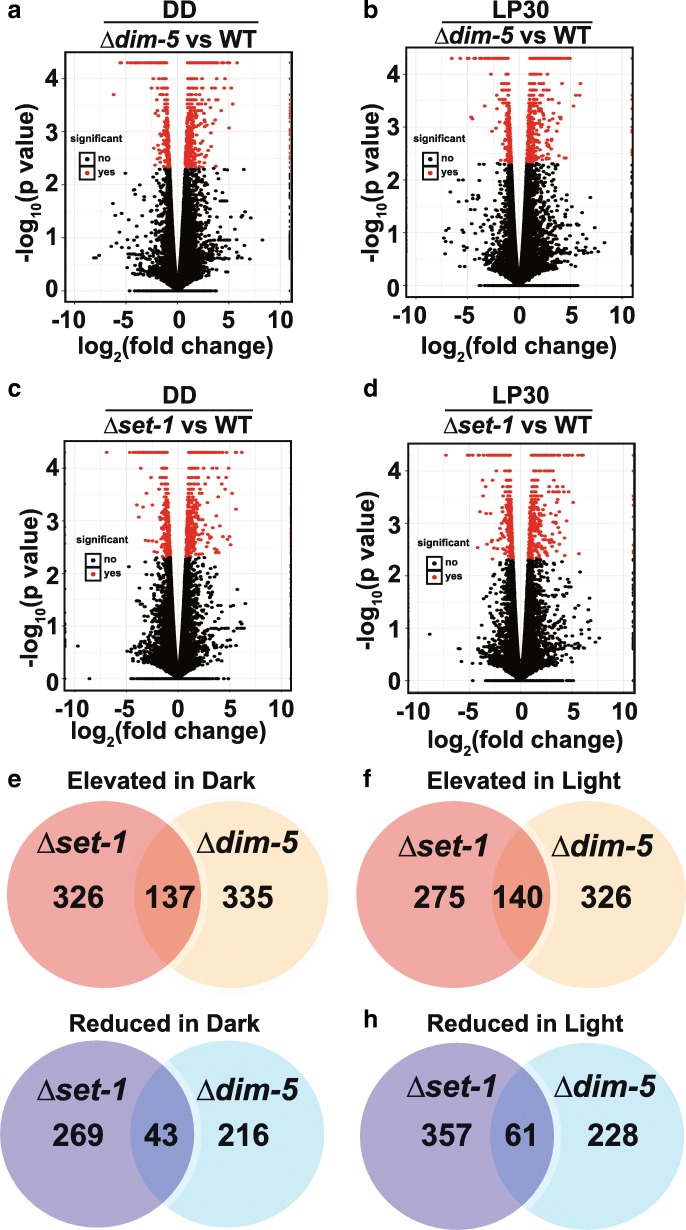
KMT1 and KMT2 are needed for activation and repression. Volcano plots indicating the expression changes in (**a**) ***∆****kmt1/****∆****dim-5* grown in the dark (DD) or (**b**) in the light. The red dots represent genes that have altered expression in the mutant relative to WT (q < 0.05). Same as in (**a**) and (**b**), except we examined expression changes in ***∆****kmt2/****∆****set-1* relative to WT in DD (c) or LP30 (**d**). (**e**-**h**) Venn diagrams show the amount of overlap among the misregulated genes. (**e**) Genes that are elevated in the dark in both ***∆****kmt2/****∆****set-1* (orange) or ***∆****kmt1/****∆****dim-5* (yellow) in DD. (**f**) Genes elevated in the light in in ***∆****kmt2/****∆****set-1* (orange) or ***∆****kmt1/****∆****dim-5* (yellow). (**g**) Genes that have reduced expression in the dark *kmt2/****∆****set-1* (dark blue) and ***∆****kmt1/****∆****dim-5* (light blue). (**h**) Genes that have reduced expression in the light in ***∆****kmt2/****∆****set-1* (dark blue) and ***∆****kmt1/****∆****dim-5* (light blue)

We next sought to better define how loss of H3K4 or H3K9 methylation changed the underlying steady-state expression in the dark and after 30 min light. We did differential analysis to find genes that had expression differences (q < 0.05) in both the dark and light for *Δkmt1/Δdim-5* and *Δkmt2/Δset-1* relative to WT. The changes in gene expression relative to WT in DD24 and LP30 are pronounced for both *Δkmt1/Δdim-5* **(**Fig. 2a & b and Additional file 5) and *Δkmt2/Δset-1* (Fig. 2c & d and Additional file 5**)**. Overall, we found that for some genes, KMT1/DIM-5 and KMT2/SET1 are needed for repression, and at other loci, they are needed for co-activation. Next, we explored the extent of overlap among the misregulated transcripts in *Δkmt1/Δdim-5* and *Δkmt2/Δset-1* and found that 137 transcripts were elevated in both *Δkmt1/Δdim-5* and *Δkmt2/Δset-1* relative to WT in the dark (Fig. 2e), while 140 transcripts are elevated in the light (Fig. 2f). In contrast, 43 transcripts had reduced expression in both *Δkmt1/Δdim-5* and *Δkmt2/Δset-1* in the dark (Fig. 2g), while 61 were reduced at LP30 (Fig. 2h). To further define the gene expression pattern among all conditions and within the mutants, we used K-means clustering. We arbitrarily clustered into 12 groups (determined empirically) reasoning this gave a good a cross-comparison of the 3 strains under both conditions (Additional file 6). Overall, many genes in *Δkmt1/Δdim-5* and *Δkmt2/Δset-1* maintained a similar pattern to WT (Clusters 1, 2, 4, 5, & 7), while others were marginally or dramatically affected in *Δkmt1/Δdim-5* or *Δkmt2/Δset-1* (Clusters 3, 6, 8–12). Specifically, cluster 8 contained light-activated transcripts that are de-repressed in both *Δkmt1/Δdim-5* and *Δkmt2/Δset-1* but remain light responsive. Cluster 9 was de-repressed in *Δkmt1/Δdim-5* while clusters 3 and 10 were de-repressed in both strains. In contrast, cluster 11 required *kmt1/dim-5* for normal expression, while cluster 6 required *kmt2/set-1* for normal expression. Of note, cluster 12 contained genes that were de-repressed in *Δkmt2/Δset-1.*

### Genome-wide distribution of H3K9me3 and H3K4me3

To further examine the connection between H3K9me3 and H3K4me3, and how these modifications influence expression, we performed H3K9me3 ChIP-seq in WT (DD and LP30) and *Δkmt2/Δset-1* (DD and LP30) using *Δkmt1/Δdim-5* DD for background subtraction. Likewise, we performed H3K4me3 ChIP-seq in WT (DD and LP30) and *Δkmt1/Δdim-5* (DD and LP30) using *Δkmt2/Δset-1* DD as background. We identified broad peaks enriched with H3K9me3 and narrow peaks with H3K4me3 using macs2 (*p* < 0.1, the macs2 cutoff used by ECNODE). The broad peak option was chosen for H3K9me3 because the vast majority of H3K9me3 occurs in constitutive heterochromatin domains that are relics of repeat induced point mutations (RIP) [33, 45]. In contrast, H3K4me3 is typically restricted to nucleosomes near the transcriptional start site and deposited in more localized regions [19, 26]. Consistent with this, we found H3K9me3 was enriched at centromeres and constitutive heterochromatin, but H3K4me3 was absent from those same regions in all conditions (data not shown).

**Fig. 3:**
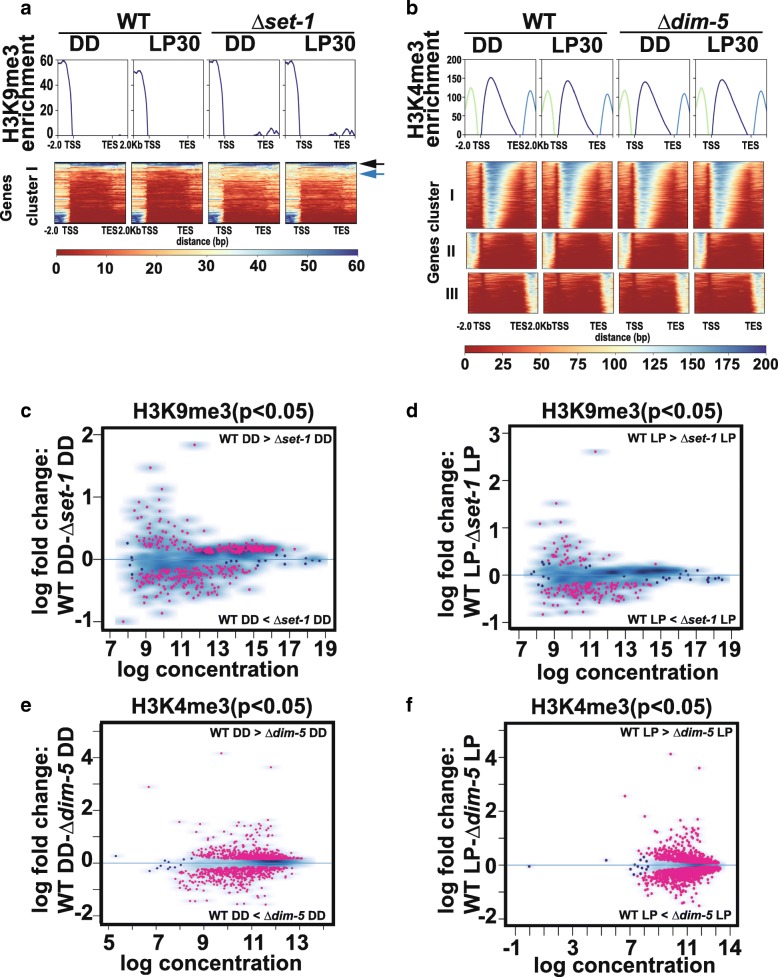
Global profile of H3K9me3 and H3K4me3**.** (**a**) The heatmaps display H3K9me3 enrichment for a subset of genes in WT and ***∆****kmt2/****∆****set-1.* The blue arrow highlights genes enriched for H3K9me3 in the gene body in both WT and ***∆****kmt2/****∆****set-1*, while the blue arrow indicates transcripts that had elevated H3K9me3 in ***∆****kmt2/****∆****set-1*. (**b**) Similar to panel (**a**), except we examine H3K4me3 in WT and ***∆****kmt1/****∆****dim-5*. Three independent clusters show the spatial distribution of H3K4me3 for transcript identified in Fig. 1a and included 2 kb upstream of TSS and downstream of TES. Clusters not shown for H3K9me3 and H3K4me3 did not have a ChIP-seq peak associated with them. (**c** & **d**) The MAplots show quantitative difference in H3K9me3 peak densities between WT and ***∆****kmt2/****∆****set-1* in (**c**) DD and (**d**) LP30. (**e**-**f**) Same as in (**c**) & (**d**) except differences in H3K4me3 peak densities were between WT and ***∆****kmt1/****∆****dim-5*. The red spots on the MA plots indicate a log fold change (*p* < 0.05)

To further examine H3K9me3 and H3K4me3 distribution, ChIP-seq peak intensities were clustered for all 21,475 transcripts identified including lincRNAs, NATs and spliced isoforms. We cluster H3K9me3 and H3K4me3 into 4 groups (determined empirically) to represent our findings and included 2 kb of DNA upstream and downstream of the transcriptional start site (TSS) and transcriptional end site (TES), respectively. As expected, H3K9me3 was largely absent from expressed genes and 2 of the clusters had no associated peaks (data not shown). However, in the other 2 clusters, H3K9me3 was present in the gene body of a small subset of loci (Fig. 3a, black arrow) and there were also peaks upstream of the TSS (Fig. 3). In total the cluster I contained 1348 transcripts. We also found H3K9me3 downstream of the TES (Additional file 7). The upstream and downstream peaks represent transcripts adjacent to constitutive heterochromatin. Consistent with a different distribution, H3K4me3 tended to cluster at nucleosomes proximal to the TSS (Fig. 3b, cluster I, 6076 transcripts). There were also instances where H3K4me3 was enriched upstream of the TSS (cluster II, 2604 transcripts) and downstream of the TES (cluster III, 2936). The second 2 clusters have H3K4me3 in upstream genes (cluster II) and downstream genes (cluster III) due to the compact nature of the Neurospora genome where most genes are within 2 kb of an adjacent gene. The remaining H3K4me3 cluster was largely devoid of H3K4me3 (data not shown). We next sought to examine the extent of H3K9me3 and H3K4me3 spreading in either *Δkmt2/Δset-1* or *Δkmt1/Δdim-5*. Alternative clustering revealed a minor trend where H3K9me3 spread into gene bodies in *Δkmt2/Δset-1*, but there was no evidence of H3K4me3 spreading in *Δkmt1/Δdim-5* (Additional file 8). The spreading of H3K9me3 into euchromatin regions in the absence of H3K4 methylation supports the notion that K4 methylation helps establish chromatin boundary elements and is consistent with the role of KMT2/SET-1 as an antisilencing factor [22].

### Interdependent relationships between H3K4me3 and H3K9me3

We next examined if there were any interdependent relationships between H3K4me3 and H3K9me3, which if present, would presumably be opposing effects. To accomplish this, we examined how H3K9me3 changed in *Δkmt2/Δset-1*, and how H3K4me3 changed in *Δkmt1/Δdim-5* relative to WT in the dark and light. We began by examining H3K9me3 in WT compared to *Δkmt2/Δset-1* in DD and LP30 (Fig. 3c & d, Additional file 9). In the dark, we found 440 H3K9me3 peaks changed between WT and *Δkmt2/Δset-1* (Fig. 3c). Among them, 199 peaks have higher H3K9me3 levels in the absence of K4 methylation, whereas 241 peaks have lower H3K9me3 levels in *Δkmt2/Δset-1*. Similar results were found in the light where 210 peaks changed between WT and *Δkmt2/Δset-1* (Fig. 3d). Among those, 165 peaks have higher H3K9me3 density in *Δkmt2/Δset-1*, compare to 44 peaks that have lower H3K9me3 density. The decrease or loss of H3K9me3 in the absence of KMT2/SET1 indicates KMT2/SET1 is required for facultative heterochromatin including *frq* (see below), but it remains to be determined if this is direct or indirect. However, much of the increase may be due to spreading of H3K9me3 into euchromatin in the absence of H3K4 methylation as determined above.

Next, we examined how H3K4me3 changed when H3K9 methylation was absent. We did similar differential analysis comparing *Δkmt1/Δdim-5* relative to WT in the dark and light. We found 1530 H3K4me3 peaks changed between WT and *Δkmt1/Δdim-5* in the dark. Among those, 537 peaks have higher H3K4me3 levels in *Δkmt1/dim5*, whereas 993 peaks have lower H3K4me3 levels (Fig. 3e, Additional file 10). In the light, a total of 2227 peaks changed intensity between WT and *Δkmt1/Δdim-5*. Among those, 1415 peaks have higher H3K4me3 density in *Δkmt1/Δdim-5* and 812 peaks had a decrease in H3K4me3 (Fig. 3f**,** Additional file 9). The changes in peak intensities for all comparisons are contained in Additional file 9.

**Fig. 4:**
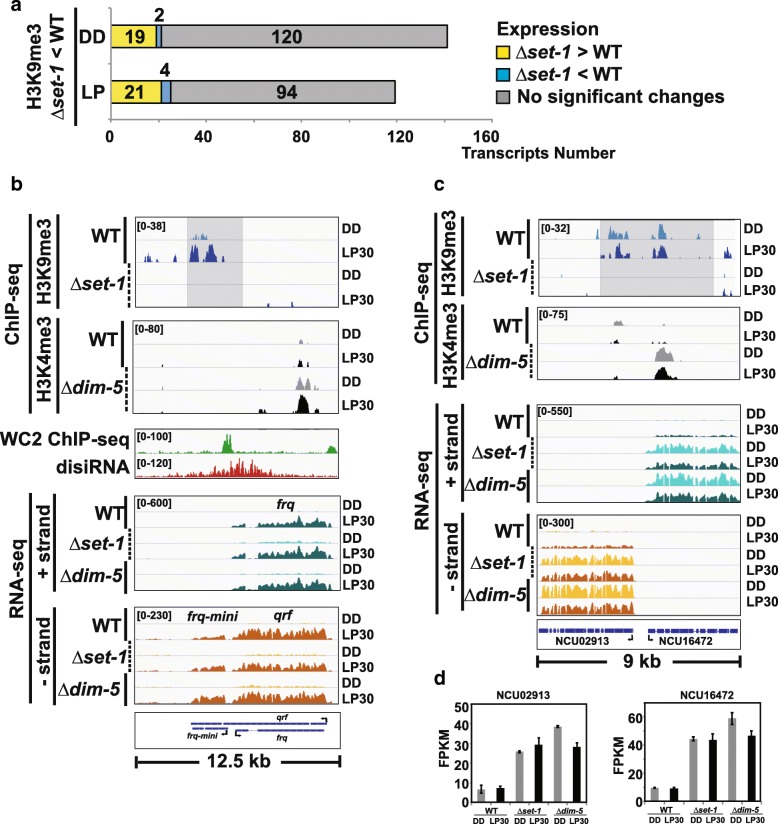
Examples where KMT2/SET1 is needed for H3K9me3. (**a**) Stacked bar plot shows the number (x-axis) of transcripts in proximity to an H3K9me3 peak that had a decreased H3K9me3 in ***∆****kmt2/****∆****set-1* (p < 0.05). Corresponding transcripts were catalogued based on whether there was a change in expression relative to WT (q < 0.05). Yellow represents elevated expression in ***∆****kmt2/****∆****set-1,* blue indicates a lower expression and grey are transcripts with no significant change. (**b**) Gene-level plot of ChIP-seq and RNA-seq of the *frq* locus. H3K9me3 peaks from the ChIP-seq done in WT and ***∆****kmt2/****∆****set-1* along with H3K4me3 peaks from ChIP performed in WT and ***∆****kmt1/****∆****dim-5.* The growth conditions (DD or LP30) are shown to the right. RNA-seq data is separated to display transcripts that originate from the plus (+) or minus (−) strand and are separated for the 3 different strains under each condition. As an added reference WC-2 ChIP-seq and disiRNA at *frq* are also shown. The bracketed numbers in the traces show the data range of the sequence alignments. (**c**) Same as in (**b**) except the diagram shows the divergently transcribed genes NCU02913 and NCU16472. (**d**) Bar plot showing the expression changes of NCU02913 and NCU16472 in WT, ***∆****kmt2/****∆****set-1* and ***∆****kmt1/****∆****dim-5* in DD and LP30

In order to examine the expression pattern of transcripts that had a decrease in H3K9me3 in *Δkmt2/Δset-1,* we extracted the peaks with a reduced H3K9me3 (log fold change ≥0.3) in *Δkmt2/Δset-1* and identified the corresponding transcripts based on proximity to the H3K9me3 peak. Overall there were 141 transcripts in the dark and 119 transcripts in the light that had a decrease in H3K9me3 when H3K4 methylation was missing (Fig. 4a). Of the 260 transcripts that had a decrease in H3K9me3 in *Δkmt2/Δset-1*, the majority had no change in expression; however, there was a small subset that appeared to be de-repressed due to loss of heterochromatin in *Δkmt2/Δset-1* (19 in the light and 21 in the dark). We classified these as genes having KMT2/SET-1-dependent heterochromatin. Also, six genes (2 in the DD and 4 in the LP) appear to have reduced expression when H3K9me3 is lost in *Δkmt2/Δset-1* (Additional file 10a-c).

We previously showed H3K4me3 functions as a repressive modification at *frq* and KMT2/SET-1 is needed for circadian negative feedback and DNA methylation at *frq* [18]*.* To gain insight into a possible mechanism of the repression, we focused on the spatial distribution of both modifications within *frq* and examined how the marks changed in the mutants relative to WT. Examination of the RNA-seq, showing the 3 known transcripts that originate from *frq* (*frq, qrf* and a small upstream transcript that spans the c-box coined *frq-mini*) and the ChIP-seq showing H3K4me3 and H3K9me3 is contained in Fig. 4b. Also included are WC-2 ChIP-seq (SRX015820) and disiRNA-seq (GSE21175) to provide more context to the chromatin regulation and spatial distribution [41, 46]. In WT, H3K9me3 was largely confined to nucleosomes near the c-box within the *frq-mini* gene body. In response to light, there was an increase in H3K9me3 that corresponded with light-activated expression of *frq-mini.* In contrast, H3K4me3 appeared restricted to the nucleosome(s) proximal to the *qrf* TSS and was not contained within the nucleosome proximal to *frq*. As predicted, we also found that H3K9me3 is completely absent in *Δkmt2/Δset-1* (*p* < 0.005). In contrast, H3K4me3 at the *qrf* TSS appeared to increase in the absence of *Δkmt1/Δdim5,* and in response to light (p < 0.005)*.* Interestingly, H3K4me3 and H3K9me3 appear spatially separated by approximately 5 kb suggesting more complex mechanism than just H3K4me3-dependent H3K9me3, unless there is a long-range chromatin contact due to gene looping. Similar to our previous report, we did not find any change in *qrf* expression; however, *frq-mini* appeared to be elevated in both *Δkmt2/Δset-*1 and *Δkmt1/Δdim-5* (Additional file 11).

In addition to *frq,* the divergently transcribed genes NCU02913 and NCU16472 also had KMT2/SET-1-dependent H3K9me3 (Fig. 4c). In WT, there were minor hints that TSS proximal nucleosomes are K4/K9 bivalent domains because H3K9me3 and H3K4me3 are both present and overlap defined regions. Further support can be found in the observation that in the absence of H3K4 methylation, H3K9me3 was lost and in the absence of H3K9 methylation, the nucleosome(s) proximal to one of the genes (NCU16472) had a substantial increase in H3K4me3 (p < 0.005). In this instance, both genes had increased expression in *Δkmt2/Δset-*1 and *Δkmt1/Δdim-5* revealing that loss of H3K9me3, either through KMT2/SET-1-dependent H3K9me3 or by deleting *kmt1/dim-5* caused de-repression (Fig. 4d).

**Fig. 5:**
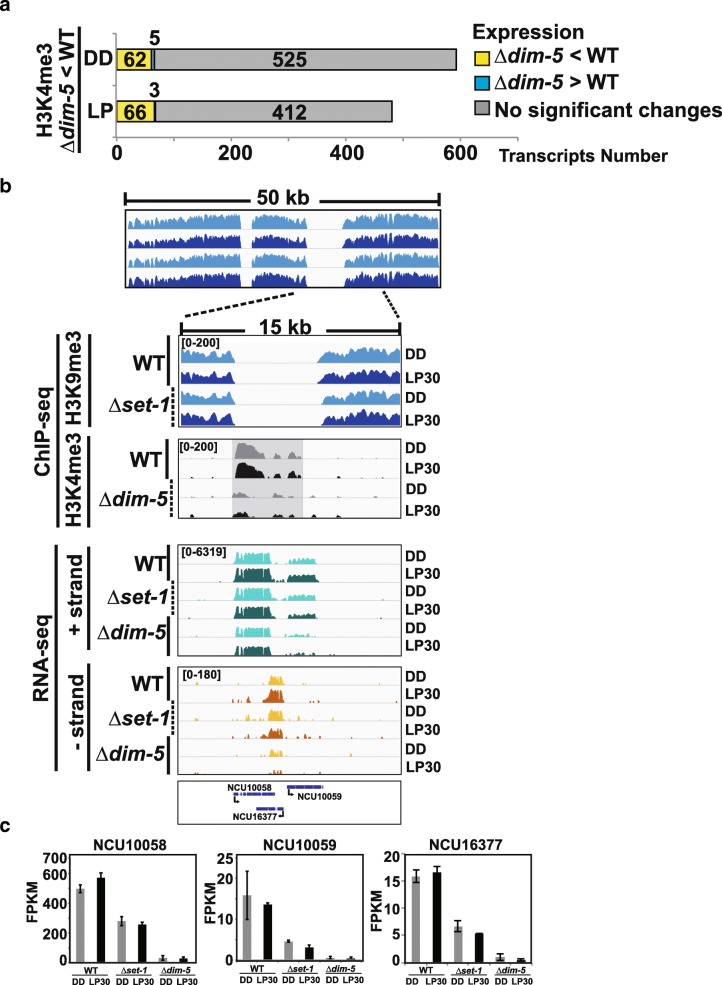
Loss of KMT1/DIM5 affects H3K4me3 distribution**.** (**a**) Stacked bar plot showing the number (x-axis) of H3K4me3-containing transcripts that have a decreased in H3K4me3 levels in *∆kmt1/∆dim-5* (p < 0.05). Transcripts are catalogued by expression changes in WT versus *∆kmt1/∆dim-5* (q < 0.05). (**b**) Representative gene-level plot of a locus that has a reduction in H3K4me3 in *∆kmt1/∆dim-5.* H3K9me3 and H3K4me3 ChIP-seq data is shown for WT and the corresponding knockouts. Transcripts originating for either the + or – strand were separated and plotted individually. (**c**) The expression level of the three genes is represented as bar plots for NCU10058, NCU10059 and NCU16377 in WT, ***∆****kmt2/****∆****set-1* and *kmt1/****∆****dim-5* comparing expression in DD (grey) versus LP30 (black)

We next examined how the reduction or loss of H3K4me3 in *Δkmt1/Δdim-5* affected expression. Overall, there were minor effects on genes that had a decrease in H3K4me3 upon loss of H3K9 methylation, and the majority showed no significant change in expression (Fig. 5a). Nevertheless, there were 62 transcripts in the dark and 66 in the light that had reduced H3K4me3 levels and lower expression in *Δkmt1/Δdim-5.* Transcripts that appeared to require KMT1/DIM-5 for normal elevated expression suggest KMT1/DIM-5 can theoretically function as a co-activator. However, this comes with major caveats because loss of H3K9me3 can cause redistribution of chromatin modifications including Histone H3 lysine 27 methylation and/or other pleiotropic effects [45, 47]; plus, genes that required KMT1/DIM-5 for expression tended to fall between regions of constitutive heterochromatin so we cannot rule out other mechanisms affecting expression. A typical example of this occurred in a region containing three genes (NCU10058, NCU10059 and NCU16377) located between 2 constitutive heterochromatin domains (Fig. 5b). In this instance, all 3 genes had a reduction in H3K4me3 levels in *Δkmt1/Δdim5* and all had a corresponding reduction in expression. These results are notable for a couple of reasons; first, expression appears lower in *Δkmt2/Δset-*1, but lowest in *Δkmt1/Δdim-5* (Fig. 5c) and second, two of the genes are convergent transcripts, but do not appear to give rise to disiRNA (not shown). Thus, we surmise the likely conclusion is that reduction in H3K4me3 and loss of expression may be due to spreading or redistribution of other chromatin modifications such as H3K27 methylation, and/or that flanking constitutive heterochromatin is necessary to maintain localized chromatin states.

**Fig. 6:**
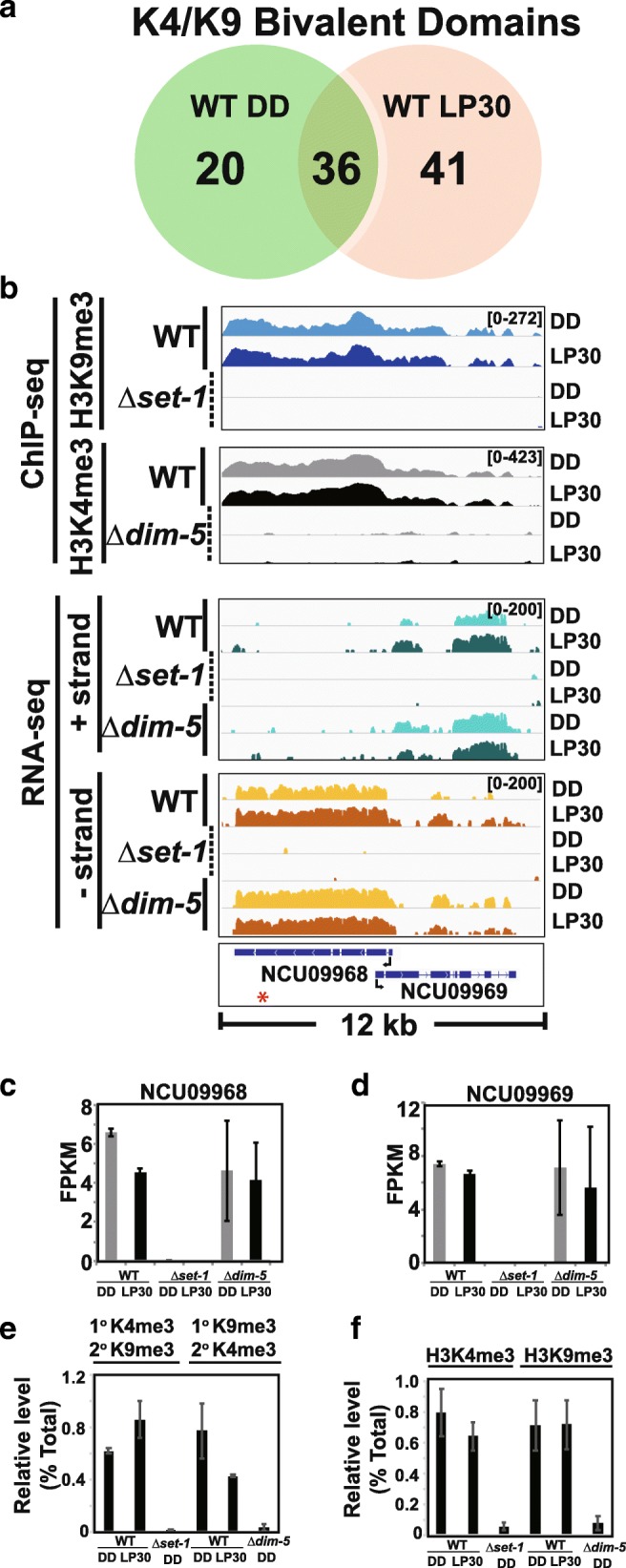
H3K4me3 and H3K9me3 can be co-dependent. (**a**). Venn diagram show presumptive K4/K9 bivalent domains in WT that contain both H3K4me3 and H3K9me3 and the extent of overlap in DD and LP30. (**b**) The gene level plot displays H3K9me3 ChIP-seq (DD, Blue and LP30, Navy) in WT and ***∆****kmt2/****∆****set-1* and H3K4me3 ChIP-seq (DD, Grey and LP30, black) in WT and ***∆****kmt1/****∆****dim-5* at divergently transcribed NCU09968 and NCU09969. Relative expression of transcripts originating from either the plus (Green) or minus (Orange) strands is shown for the 3 strains. NCU09969 also has an antisense transcript(s) identified by SringTie (not drawn). Expression level of (**c**) NCU09968 and (**d**) NCU09969 are shown as bar plots in WT, *kmt2/****∆****set-1* and *kmt1/****∆****dim-5* for DD (grey) and LP30 (black). (**e**) Reciprocal sequential ChIP (re-ChIP) in WT from 3 independent biological replicates. The first antibody (1^o^) and second antibody (2^o^) used in the re-ChIP are shown above the bar plot and the amount recovered from the re-ChIP was determined by determine by qPCR and represented as a percentage of the input (Relative level, % Total). The red asterisk on the gene-level plots show the location of the oligo used for re-ChIP (**f**) For each re-ChIP experiment, we removed one tenth of the primary ChIP and measured the amount recovered as a control using the antibodies listed above the graph

We next focused on genes with a co-dependent and overlapping relationship between H3K9me3 and H3K4me3. In total, we found 56 loci that contained both modifications in the dark and 77 after LP30 (Additional file 12). 36 of these loci were shared under both environmental conditions (Fig. 6a). In certain instances, H3K4me3 was lost without H3K9 methylation and H3K9me3 was lost without H3K4 methylation. One example occurred in a transposon containing NCU09968 and *sly*-1 (NCU09969), which incidentally also appears to be a clock-controlled ADV-1 target gene (Fig. 6b) [48–50]. Both H3K9me3 and H3K4me3 are contained within the gene body of NCU09968 and spread into *sly-1* and both modifications are dependent on one another. Interestingly, the expression of the two divergently transcribed transcripts was only affected in the absence of KMT2/SET-1 (Fig. 6c and d). To confirm the existence of K4/K9 bivalent domains, we performed sequential ChIP (re-ChIP) under reciprocal conditions using either H3K4me3 or H3K9me3 antibodies first (1^o^), followed by the opposing antibody in the re-ChIP (2^o^) (Fig. 6e). As an added control, we took 1/10 the first ChIP to monitor recovery (Fig. 6f). Another instance occurred at a centromeric gene on supercontig 12.2 (chromosome II) (Additional file 13). In this instance, only the heterochromatin in the gene body that contained both H3K9me3 and H3K4me3 had the co-dependent relationship and the nearby constitutive heterochromatin was not affected. As with NCU09969 and *sly-1*, expression of NCU16628 was dependent on KMT2/SET-1.

**Fig. 7:**
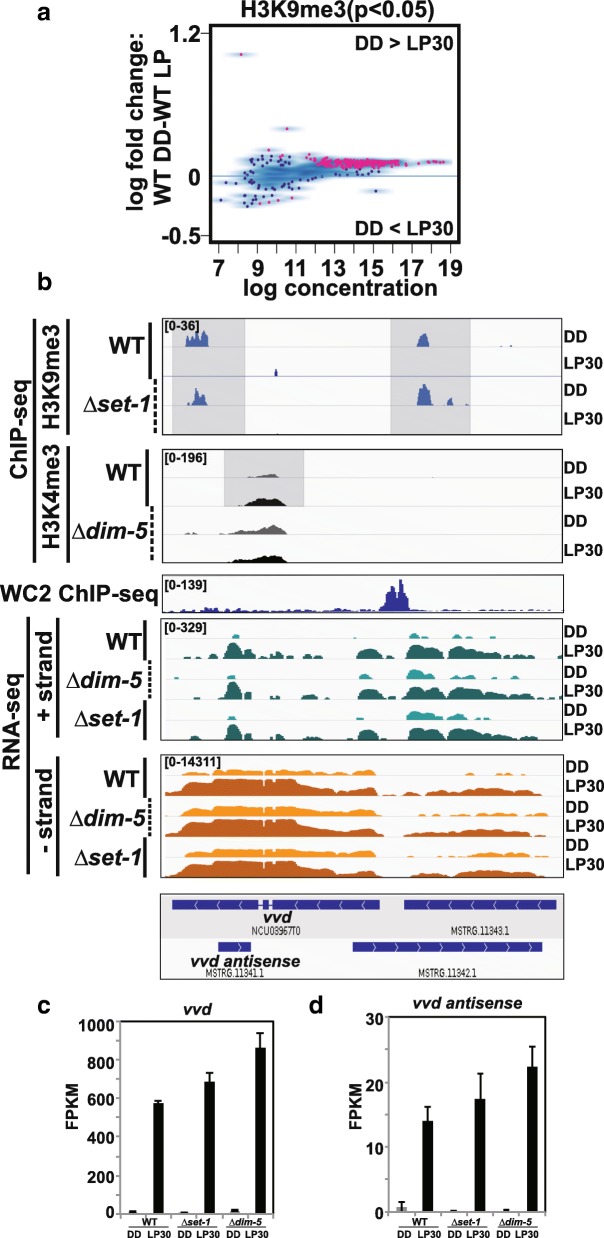
Effects of light on H3K9me3**.** (**a**) The MA plots show quantitative difference (p < 0.05) in H3K9me3 peak densities between WT DD and WT LP30. (**b**) Gene-level plot of ChIP-seq and RNA-seq of the *vvd* locus. H3K9me3 peaks from the ChIP-seq done in WT and ***∆****kmt2/****∆****set-1* along with H3K4me3 peaks from ChIP performed in WT and ***∆****kmt1/****∆****dim-5.* The growth conditions (DD or LP30) are shown to the right. RNA-seq data indicating transcripts that originate from the plus (+) or minus (−) strand are separated for the 3 different strains under the two conditions. As an aided reference WC-2 ChIP-seq is also shown. (**c**) Bar plot showing the expression changes of *vvd* and *vvd antisense* in WT, ***∆****kmt2/****∆****set-1* and ***∆****kmt1/****∆****dim-5* in DD and LP30

Loss of H3K9me3 causes heightened circadian-regulated conidia formation similar to the *ras-1*^*bd*^ mutant and *Δkmt1/Δdim-*5 has a synthetic effect with *ras-1*^*bd*^ [17]. In Neurospora, conidia development is a light-activated process, so we wanted to examine changes in H3K9me3 in response to light in WT**.** We found that 244 loci had a reduction in H3K9me3 after 30 min light, whereas only 4 peaks (including *frq*) had an increase in H3K9me3 after 30 min light in WT (Fig. 7a and Additional file 14**).** A specific example of light-activated loss in H3K9me3 occurred at *vvd,* which is a light-activated gene necessary for photoadaptation (Fig. 7b). Inspection of *vvd* revealed some unexpected findings, including the observation that *vvd* contains a light-activated NAT and there are two upstream overlapping lincRNAs that form a sense-antisense pair. As reported previously, *vvd* appeared to have an increase in light-activated expression in the absence of *Δkmt2/Δset-*1 or *Δkmt1/Δdim-*5 (Fig. 7c & d). Upon light activation, H3K9me3 is lost and there is a small increase in H3K4me3. Of note, H3K9me3 was unaffected by *Δkmt2/Δset-1* at *vvd*. In addition, there were 5 loci where H3K9me3 changed in response to light in *Δkmt2/Δset-*1 (Additional file 15a). In these instances, 4 peaks had a decrease in H3K9me3 (Additional file 15 b) while 1 peak had an increase in H3K9me3 (Additional file 15c)**.**

Finally, we examined how light affects H3K4me3 in WT and found 760 H3K4me3 peaks showed a reduction in response to light whereas 31 H3K4me3 peaks increased (Additional file 16). The finding that more transcripts lost H3K4me3 in response to light versus transcripts that gained H3K4me3 is counter to the notion that H3K4me3 is solely a mark for actively transcribed genes and may be more reflective of transcriptional memory. However, we cannot rule out that H3K4me3 nucleosomes are being disassembled to make room for transcriptional machinery, or that the relatively long 30-min light treatment caused the H3K4me3 to revert to steady-state levels. Regardless of the cause, it is clear H3K4me3, and for that matter H3K9me3, play more complex roles in transcriptional regulation than current paradigms suggest.

## Discussion

In this article, we performed a comprehensive analysis to understand how H3K9me3 and H3K4me3 impact one another and how they affect gene expression. Initially, there were some a priori assumptions, which didn’t entirely pan out as expected and instead there was a more complicated interplay between the modifications. For example, at the start, it seemed reasonable to assume that loss of KMT2/SET-1 would predominantly manifest as a defect in light-activated gene expression due to the widely-accepted premise that H3K4me3 is a mark for activation (or at the very least a mark for actively transcribed genes in euchromatin). Instead, we found that SET1 is needed for both expression and repression. In addition, we found that H3K4me3 tended to decrease after 30 min in the light (data not shown); a time when adaptation has solidly commenced, so it is possible the activating H3K4me3 modifications are being removed. It is also plausible that the reduction in H3K4me3 follows a general trend where the nucleosome density is decreasing at actively transcribed genes, which is consistent with the finding that nucleosome occupancy proximal to regions of light-activated genes decrease in response to light [12]. In contrast to KMT2/SET-1-dependent H3K4 methylation, we expected to find mostly de-repression in the absence of H3K9me3 because although H3K9me3 is widespread in Neurospora, it is largely restricted to constitutive heterochromatin domains devoid of protein coding genes, but a number of genes are de-repressed in *Δkmt1/Δdim-5* due to the redistribution of H3K27 methylation [47]. Consistent with this, we found that a number of genes are de-repressed in *Δkmt1/Δdim-5,* but we also found that KMT1/DIM-5 appears to be needed for light-activated expression (Fig. 1c). However, the extent to which these changes are pleiotropic need further examination, because even though we found numerous genes with H3K9me3, only a small subset had altered expression in the corresponding deletion strain. Thus, the misregulation that occurs in *Δkmt1/Δdim-5* is potentially due, at least in some instances, to redistribution of other modifications like re-localization of H3K27me3 [45, 47].

The initial premise of this work was rooted in the finding that H3K4 methylation, which is normally excluded from regions containing methylated DNA, is required for DNA methylation at *frq* [18]. We were hopeful that these experiments would illuminate why H3K4me3 is needed for DNA methylation at *frq,* and potentially find other loci, but after analysis of the data, a definitive conclusion is still lacking*.* However, the data did reveal that H3K9me3 was completely absent from *frq* in *Δkmt2/Δset-1,* confirming the requirement for KMT2/SET-1. The actual mechanism of how this occurs is confounding in part due to the spatial distribution of H3K4me3 and H3K9me3 within *frq*. We found that H3K4me3 was predominantly localized in a region proximal to the TSS in *qrf*, while H3K9me3 was restricted to nucleosome surrounding the c-box (Fig. 4b). Based on the need for *qrf* in facultative heterochromatin at *frq* [37], the obvious answer would suggest that loss of H3K4me3 at *qrf* might effect *qrf* expression; however, as previously reported, *qrf* expression was not altered in *kmt1/set-1* knockout [18]. It is also possible the KMT2/SET-1-dependent heterochromatin at *frq* occurs analogous to silencing of retrotransposons in *S. pombe*, which requires SET1, but appears to be independent of H3K4 methylation [23, 24]. Some ancillary support for this can be found in the region containing *Sly-1* and *NCU09968*, which we identified as a K4/K9 bivalent domain (Fig. 6)*.* This domain is believed to be a transposon in the WT parent strain (FGSC2489) used in this study [48], but it also appears to be circadian regulated [49] and a target of ADV-1 [50]. However, in this instance both modifications are present and contained on the same nucleosome, but expression is only dependent on H3K4me3. Another possible explanation is that the KMT2/SET-1 dependent facultative heterochromatin may be caused by secondary affects. However, when we examined the expression of known components of DCDC, DRDM, or RNAi components, we did not find any major defects, nor did we observe any other global changes in constitutive heterochromatin. Support for a direct effect comes from findings that other loci displayed similar co-dependent requirements and thus *frq* is not entirely unique. Whether or not mono- or di-methylation of H3K4, and not H3K4me3, are the requisite modifications for H3K9me3 needs to be examined further. For example, one could speculate that H3K4me1 and H3K4me2 (or other modifications requiring KMT2/SET-1) are missing in *Δkmt2/Δset-1* and these modification(s) overlap with the peak in H3K9me3. An example of this hypothesis would posit that H3K4me1, which tends to accumulate at the 3′ end of genes, would be enriched at the 3′ end of *qrf*, and this is close to the H3K9me3 peaks. However, this is pure speculation at this juncture and more comprehensive series of studies are needed, because even if H3K4me1 is co-localized and missing; it does not reveal the mechanism. Regardless, is remains clear that at *frq,* and 260 other loci, KMT2/SET-1 influences H3K9me3-mediated facultative heterochromatin and in some instances H3K4me3 and H3K9me3 appear as bivalent chromatin domains [51].

As it pertains to K4/K9 bivalent chromatin domains, Neurospora is uniquely suited to further define their role in regulation because KMT1/DIM-5 and KMT2/SET-1 are the only H3K9 and H3K4 methyltransferases and neither is essential for viability so in theory, dissecting the importance of each modification in bivalent domains should be straightforward. However, it is far more complicated than meets the eye, because in certain instances, these modifications were dependent on one another and in many cases, loss of either didn’t dramatically affect the expression of the underlying transcripts. When one considers there is also redistribution of other modifications, the system becomes sufficiently complex and would likely yield inappropriate conclusions. Thus, the only thing we can infer is that H3K4me3/H3K9me3 bivalent domains represent functional states where the modifications may be reliant on one another to establish the appropriate transcriptional program and may not necessarily be in a poised state ready for activation or repression as proposed for K4/K27 bivalent domains [52]. In addition, other combinations of modifications are likely contained within the nucleosome, beyond just H3K4me3 and H3K9me3, and these will likely contribute to the transcriptional program. For example, multivalent chromatin readers may not necessarily discern if the full-compliment of modifications are present to maintain the gene in a repressed or activated state. Whether or not this extends to classical bivalent domains that contain H3K4me3 and H3K27me3 should be considered, because the data presented here seems to suggest it is more complicated than initially perceived.

As a final note, there are less than 50 convergent loci in Neurospora that contain disiRNAs and DNA methylation [39, 41] and in this report we found 2444 transcripts that contain NATs, which greatly increases the number of convergent overlapping transcripts. However, only a small subset contains DNA methylation and H3K9me3. Given that H3K9me3 is needed for DNA methylation [53], the majority of convergent transcripts do not give rise to disiRNA-mediated DNA methylation. So ultimately, convergent transcripts are not universal in their ability to generate heterochromatin and in this instance, *frq* and the other disiRNA loci, appear to be special cases with unique epigenetic regulation.

## Conclusions

Sensing and responding to light provide organisms an adaptive advantage, in part by altering gene expression. The complement of light-activated genes in model organisms is largely known, and mechanisms of activation and photoadaptation are well-defined. To further understand how light alters post translation modifications to chromatin, we performed a comprehensive analysis looking at H3K4me3 and H3K9me3. Using a combination of RNA-seq and ChIP-seq, we found that H3K4 methylation and H3K9 methylation play an important but restricted role in light-activated gene expression. Both modifying enzymes need to be present for normal compliment of genes to be light-activated and repressed. In addition, we found KMT2/SET-1-dependent heterochromatin and H3K4me3/H3K9me3 bivalent domains that appear to function in a complex intertwined manner that merits further research but not necessarily part of the light response.

## Methods

### Strains and growth conditions

The strains used in this report are WT (FGSC 2489), *Δkmt2/Δset-*1 (XB140–10) and *Δkmt1/Δdim5* (XB18–11) and were described previously [17, 18]. Briefly, *Δkmt2/Δset-*1 and *Δkmt1/Δdim5* were made by the Neurospora Knockout Consortium and obtained from the Fungal Genetics center then backcrossed to FGSC2489. Neurospora was grown as follows; conidia were inoculated in 2% liquid culture media (2% LCM) consisting of 1X Vogel’s salts, 2% glucose, 0.17% arginine and grown in 100 mm Petri dish overnight at 30 °C to generate mycelia mats. Plugs were cut from the mycelia and used to inoculate flasks containing 100 ml of 2% LCM and grown for a total of 2 days at 25 °C. For DD24, cultures were grown in the light for 24 h then transferred to constant dark for 24 h and harvested. The LP30 samples were grown in a similar fashion but just prior to harvesting, the cultures were placed in constant light for 30 min. Depending on the experiment the tissue was either immediately snap frozen or crosslinked with 1% formaldehyde.

### RNA-seq sample preparation

Frozen tissue from WT, *Δkmt2/Δset-*1 and *Δkmt1/Δdim-5* grown in constant darkness for 24 h or subject to a 30-min light pulse. A small fraction of the ground mycelia was used to extract total RNA using Trizol (Invitrogen) following the manufacturer’s instructions. The total RNA from 2 biological replicates was sent to the Columbia Genome Center for library preparation and RNA-seq. Ribosomal RNAs were depleted and the remaining RNA was for cDNA library preparation using the TrueSeq library preparation kit version 2. A total of 60 million 100 bp paired-ended reads were sequenced on the Illumina 2500 instrument. The RNA-seq is deposited in Gene Expression Omnibus (GEO) with the accession number, GSE121356.

### RNA-seq analysis

We used the HISAT2 (version 2.1.0) and StringTie (version 1.3.3b) [42] for mapping and transcripts discovery. The 100 bp pair-end reads were mapped to the Neurospora NC12 reference genome using the existing GTF file as a guide with the default settings [54]. The resulting Neurospora transcriptome for each sample and time point was assembled and merged with the existing GTF file using StringTie to generate a new GTF file containing novel transcripts. Gffcompare (version v0.10.1) was used with the 2 gtf files to determine how many assembled transcripts matched annotated genes. NATs and lincRNAs determined based on class code; NATs were called if the class code was x or s, then filtered by i and o to determine overlap and direction; lincRNA were called if the transcript had a class code:u. Next, we used Cuffdiff (version v2.2.1) and cummerbund (version 2.20.0) [55, 56] for differential expression and statistical analysis. Genes that had a change in expression between light and dark or between strains under identical conditions were determined using the getSig function in CummeRbund (q < 0.05) [57]. Transcripts visualization was done using Integrative Genomics Viewer (IGV) (version 2.3.94) [58].

### Chromatin immunoprecipitation

The ChIP experiments were performed on WT, *Δkmt2/Δset-1* and *Δkmt1/Δdim-5* at DD24 and LP30 of two biological replicates. The ChIP experiments followed our general laboratory protocol. Tissue was crosslinked with 1% formaldehyde for 10 min then quenched with 0.1 M Glycine for 10 min. Crosslinked tissue was ground with a mortar and pestle, and the lysates were suspended in 10 mL of ChIP lysis buffer [0.05 M Hepes (pH 7.4), 0.15 M NaCl, 0.001 M EDTA, 1% Triton X-100, 0.1% SDS] containing protease inhibitors (2.0 μg/mL leupeptin, 2.0 μg/mL pepstatin A, 1.0 mM PMSF). Complete cell lysis and gross chromatin shearing was achieved by sonicating 2 times at 20% power with a microtip. The resulting lysates were cleared of cellular debris by centrifugation at 2000×g for 10 min. 200 μl of supernatant was transferred to a 1.5 ml polystyrene tube and the chromatin was sheared to an average size of 500 bp using a Misonix cup sonicator 6–8 times at 20% power. The ChIPs were performed with antibodies specific to H3K9me3 (Abcam #ab8898) and H3K4me3 (Abcam #ab8580). Each ChIP sample contained approximately 10 μg of lysate and 0.025 μg antibody prebound to Protein A magnetic beads. The antibody, lysates and beads were incubated overnight at 4 °C and washed 4 times with RIPA buffer then eluted with 0.1 M sodium bicarbonate containing 1%SDS. The crosslinks were reversed by heating at 65 °C and the protein was removed by the addition of proteinase K. The DNA was further purified by phenol chloroform extraction and ethanol precipitation. The purified DNA was sent to Beijing Genome Institute (BGI) for library preparation and sequencing. The samples were sequenced to a depth of 50 million 50-bp single-end read. The ChIP-seq data is deposited in GEO and can be found under GSE121356.

### ChIP-seq data analysis

ChIP-seq reads were mapped to NC12 using Burrows-Wheeler Aligner (BWA) (version 0.7.10) [59]. The resulting Bam files were processed using macs2 call peak function [60]. For H3K9me3, we used the broad peaks options with the H3K9me3 ChIP from *Δkmt1/Δdim-5* DD as the control file for background subtraction. For H3K4me3 ChIP, we used the default setting and the H3K4me3 ChIP on *Δkmt2/Δset-1* as the control file. Next, we used Diffbind (version 2.6.6) [61] to determine H3K9me3 and H3K4me3 enrichment between the different strains and conditions and visualized the data with the MA plot (dba.plotMA) function (*p* < 0.05). ChIPseeker (version 1.14.1) [62] was used to examine coverage of H3K9me3 and H3K4me3 over each chromosome using the *covplot* function (not shown). The *TagHeatmap* function was used to generate the heatmap relative to the TSS plus or minus 2000 bp. In addition, we used deepTools2 [63] plotHeatmap function to create additional heatmaps. RnaChipIntegrator was used to determine genes that are close to ChIP-seq regions using the -edge = both function within 2 kb of the TSS and TES. We used *bedtools intersect* (version v.2.25.0) [64] to determine the overlap of H3K4me3 and H3K9me3.

### Sequential ChIP

The sequential ChIP followed our standard ChIP protocol described above with minor modifications. First, the H3K4me3 and H3K9me3 antibodies were covalently attached to BioMag carboxyl microparticles (Bangs Laboratories) using EDAC following manufacturer’s guidelines. After coupling, beads were titrated to find the optimal antibody-bead to lysate ratio. The primary ChIP used 10 times the normal amount used in a standard ChIP (approximately 50 μg of lysate) and 50 μl beads. After the initial ChIP, the chromatin was eluted with 2 × 25 μl (0.1 M NaHCO_3_, 1.0% SDS) by heating at 37 °C for 10 min. The pH of eluates was adjusted to near neutral pH by the addition of 6 μl of 1.0 M Tris pH 6.5. The SDS was lowered to 0.1% for the second ChIP by the addition of 10 times the eluate volume of ChIP Lysis Buffer with no SDS (500 μl). At this stage, 1/10 the initial ChIP was removed and the remaining was subjected to a second ChIP with 10 μl beads containing the second antibody. For the sequential ChIP we performed reciprocal reactions to fully validate the findings; In one re-CHIP, H3K4me3 was used first and H3K9me3 second. Simultaneously, separate ChIP was performed using H3K9me3 first and H3K4me3 second. In total, the sequential ChIP was performed 3 independent times on 3 separate biological replicates in reciprocal duplicates.

## Additional files

Additional file 1: (a) Schematic representation of the transcript discovery pipeline used in this study. After StringTie merge, we identified 21,475 transcripts at the isoforms level using the default settings in HISAT2 and StringTie. Transcripts expression differences were identified using Cuffdiff and further analysis was performed using CummeRbund. Gffcompare was used to classify newly identified transcripts relative to the reference annotation NC12. (b) Multidimensional scaling of the RNA-seq samples reveal that the underlying mutations have a larger effect than light treatment. M1: Dimension 1, M2: Dimension 2. (PDF 389 kb)

Additional file 2: Variation among transcript types in *∆kmt2/∆set-1* and *∆kmt1/∆dim-5*. Violin plot depicting the expression levels (FPKM, log2) of transcripts belonging to existing annotated transcripts, NATs and lincRNAs in (a) *kmt2/∆set-1* (b) *kmt1/∆dim-5*. (PDF 721 kb)

Additional file 3: Table of Light-responsive genes in WT, *∆kmt2/∆set-1* and *∆kmt1/∆dim-5. (XLS 285 kb)*

Additional file 4: Hierarchical clustering of light regulated genes in *∆kmt2/∆set-1* and *∆kmt1/∆dim-5*. (a) Heatmap showing the clustering of genes that are differentially expressed in *∆kmt1/∆dim-5* DD versus LP30 and compared to same genes in WT and *∆kmt2/∆set-1.* The expression levels are log2 fold change (q < 0.05). (b) Same as in A except clustering was done in *∆kmt2/∆set-1* DD versus LP30. Genes in Group A are light activated and Group B includes genes that are light repressed. Changes in expression between the two conditions are displayed with range [− 10,10], with levels above and below the mean shown in yellow or blue. (c) Bar plot depicting the number of different transcripts categorized as an existing annotated gene, lincRNA, NAT or other that were light activated in WT, *∆kmt1/∆dim-5* and *∆kmt2/∆set-1*. The x-axis is the gene number and the y-axis is the strain. (PDF 483 kb)

Additional file 5: Table of genes showing a transcriptional deficit in *∆kmt2/∆set-1* and *∆kmt1/∆dim-5* relative to WT under dark and light conditions. (XLS 768 kb)

Additional file 6: Gene expression clusters of WT, *∆kmt1/∆dim-5* and *∆kmt2/∆set-1.* Gene expression patterns were clustered into 12 groups based on the expression profile. The colored lines represent the pattern for each gene, and the black lines represent the median of all the genes in a given cluster. (PDF 1434 kb)

Additional file 7: The heatmaps display ChIP enrichment of H3K9me3 in WT and ***∆****kmt2/∆set-1*. Regions included 2 kb upstream of TSS and downstream of TES. In this specific cluster, H3K9me3 is found downstream of the TES in a small subset of genes adjacent to constitutive heterochromatin. (PDF 665 kb)

Additional file 8: H3K9me3 spreading in *kmt2/∆set-1* strain. Heatmap display signal distribution for (A) H3K9me3 (B) H4K4me3 density plotted in a 2-kb windows centered on the TSS. The curly bracket(s) in panel A indicate the extent of H3K9me3 spreading in ***∆****kmt2/∆set-1*. (C) Gene-level plot of a 236 kb region on chromosome VII (supercontig 12.7) showing H3K9me3 ChIP-seq (DD Blue and LP30 Navy) for the WT and ***∆****kmt2/∆set-1* and H3K4me3 ChIP-seq (DD Grey and LP30 black) for the WT and ***∆****kmt1/∆dim-5*. The shaded boxes highlight representative examples of H3K9me3 spreading into euchromatic regions in ***∆****kmt2/∆set-1*. (PDF 868 kb)

Additional file 9: Differences and location of H3K9me3 and H3K4me3 peak intensities in WT relative to the contrasting deletion strain in the dark and light. (XLS 684 kb)

Additional file 10: Additional loci that have KMT2/SET-1-dependent heterochromatin. Gene-level diagram of H3K9me3 ChIP-seq (DD Blue and LP30 Navy) in WT and ***∆****kmt2/∆set-1,* and H3K4me3 ChIP-seq (DD Grey and LP30 black) in WT and ***∆****kmt1/∆dim-5* for a presumptive lincRNA, (a) MSTRG.11857 and (b) NCU03747. It is clear from the traces that both have a significant decrease in H3K9me3 (*p* < 0.05) in ***∆****kmt2/∆set-1*. (c) Expression bar plot of MSTRG.11856 and NCU03747, which have a decrease in expression when KMT2/SET-1-dependent heterochromatin is lost. (PDF 402 kb)

Additional file 11: Expression of transcripts arising from the *frq* locus. FPKM values for (a) *frq*, (b) *qrf* and (c) *frq-mini* are shown as bar plots in WT, *kmt2/****∆****set-1* and *kmt1/****∆****dim-5* for DD (grey) and LP30 (black). (PDF 388 kb)

Additional file 12: Location of presumptive bivalent domains containing both H3K9me3 and H3K4me3. (XLS 55 kb)

Additional file 13: Reciprocal dependence of H3K9me3 and H3K4me3. Gene level plot shows a centromeric gene on Chromosome II (supercontig 12.2). The IGV diagram displays H3K9me3 ChIP-seq (DD Blue and LP30 Navy) in WT and *∆kmt2/∆set-1* and H3K4me3 ChIP-seq (DD Grey and LP30 black) in WT and *∆kmt1/∆dim-5*. The corresponding RNA-seq traces are also shown for NCU16528. (PDF 427 kb)

Additional file 14: Genome location of H3K9me3 peaks that have a change in intensity in response to light. (XLSX 27 kb)

Additional file 15: Changes in H3K9me3 in response to light in *∆kmt2/∆set-1.* (a) Quantitative difference in H3K9me3 levels from the ChIP-seq in *∆kmt2/∆set-1* upon light exposure. The MA plot shows log fold change (p < 0.05) in enrichment in DD (log fold change > 0) or LP (log fold change < 0). (b) IGV diagrams of 4 genes that had a decrease in H3K9me3 in *∆kmt2/****∆****set-1* in response to light (p < 0.05) (c) IGV diagram of NCU05133 which had an increase in H3K9me3 in *∆kmt2/****∆****set-1* (p < 0.05) in response to light. (PDF 543 kb)

Additional file 16: Light induced changed in H3K4me3 in WT. Quantitative difference in H3K4me3 levels in WT DD versus WT LP30. The MA plot shows increase in H3K4me3 in DD (log fold change > 0) or in response to light (log fold change < 0). Spots shown in red have a *p* < 0.05. (PDF 449 kb)

## Abbreviations

ChIP-seq: Chromatin Immunoprecipitation with DNA sequencing
*frq*: *frequency*
H3K4me3: histone H3 lysine 4 trimethyation
H3K9me3: histone H3 lysine 9 trimethylation
KMT1/DIM-5: Histone H3 lysine 9 methyltransferase/Defective in DNA methylation 5
KMT2/SET-1: Histone H3 lysine 4 methyltransferase
RNA-seq: RNA sequencing
*vvd*: *vivid*
