# Supplementary figures and images for: Histone H3 lysine 4 methyltransferase is required for facultative heterochromatin at specific loci
Source: BMC Genomics. 2019 May 8;20:350. doi: 10.1186/s12864-019-5729-7 (PMC6505117; doi:10.1186/s12864-019-5729-7)

**a**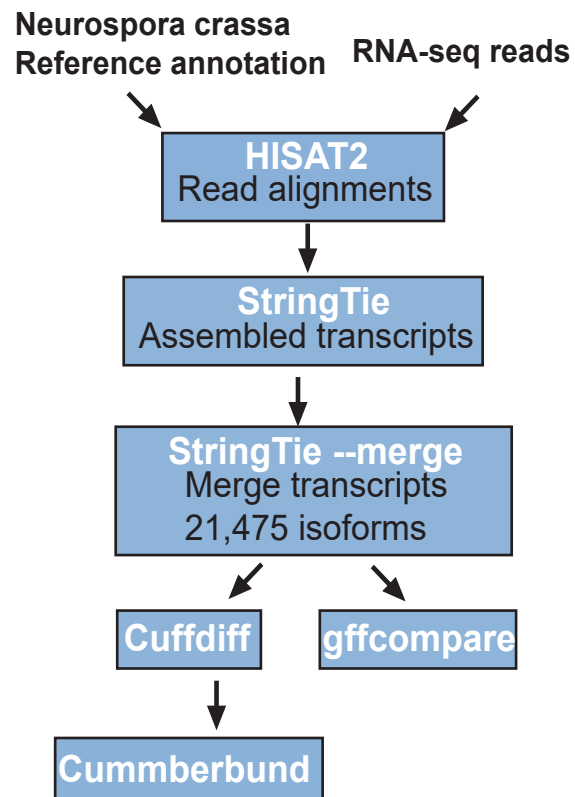**b**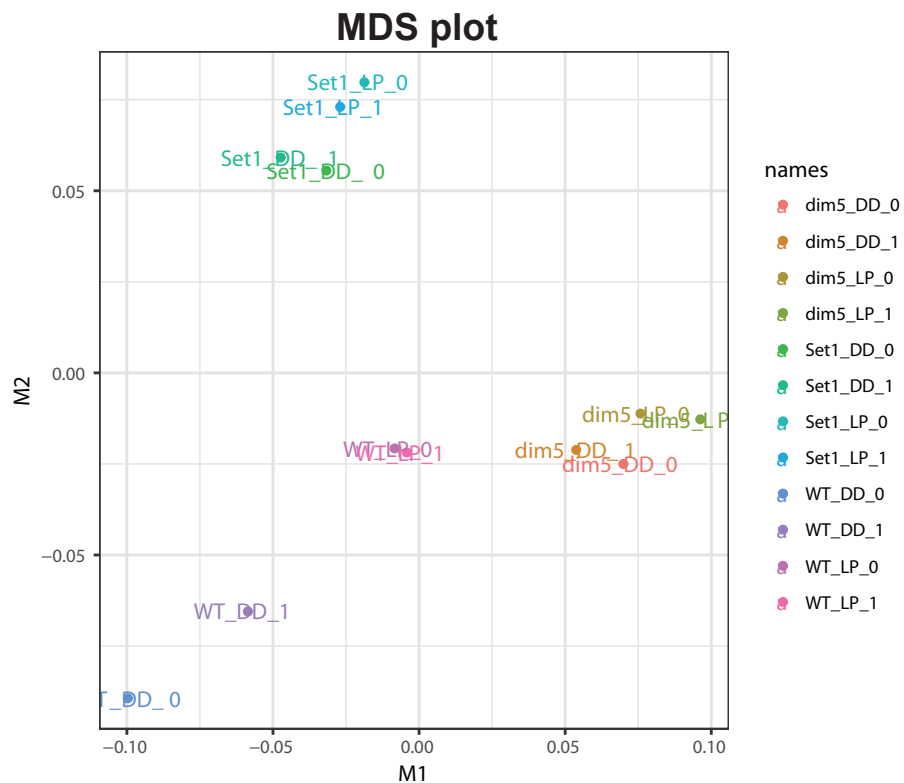

Supplement: Supplementary file 1 — (a) Schematic representation of the transcript discovery pipeline used in this study. After StringTie merge, we identified 21,475 transcripts at the isoforms level using the default settings in HISAT2 and StringTie. Transcripts expression differences were identified using Cuffdiff and further analysis was performed using CummeRbund. Gffcompare was used to classify newly identified transcripts relative to the reference annotation NC12. (b) Multidimensional scaling of the RNA-seq samples reveal that the underlying mutations have a larger effect than light treatment. M1: Dimension 1, M2: Dimension 2. (PDF 389 kb) [file 12864_2019_5729_MOESM1_ESM.pdf]

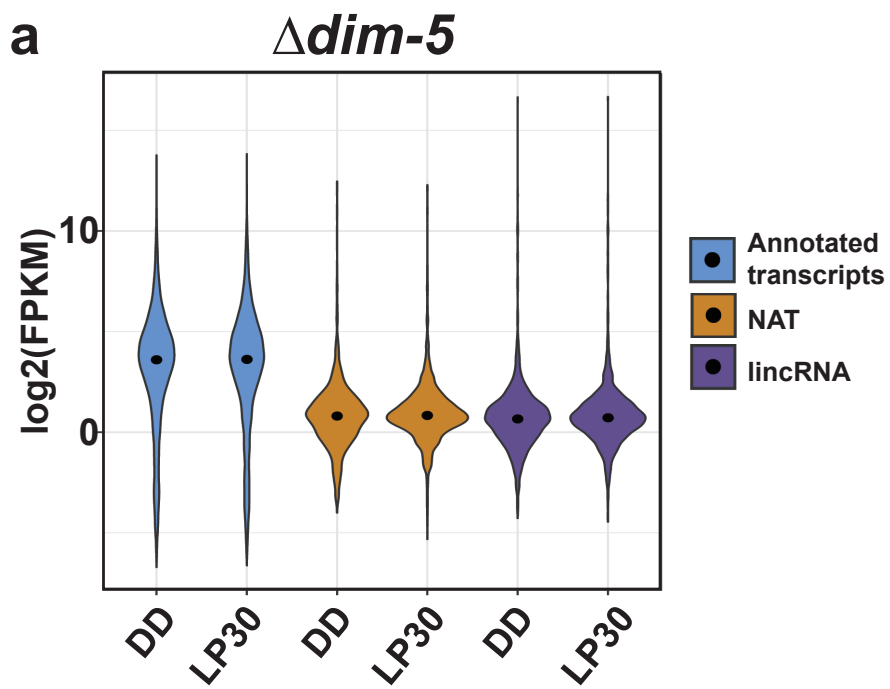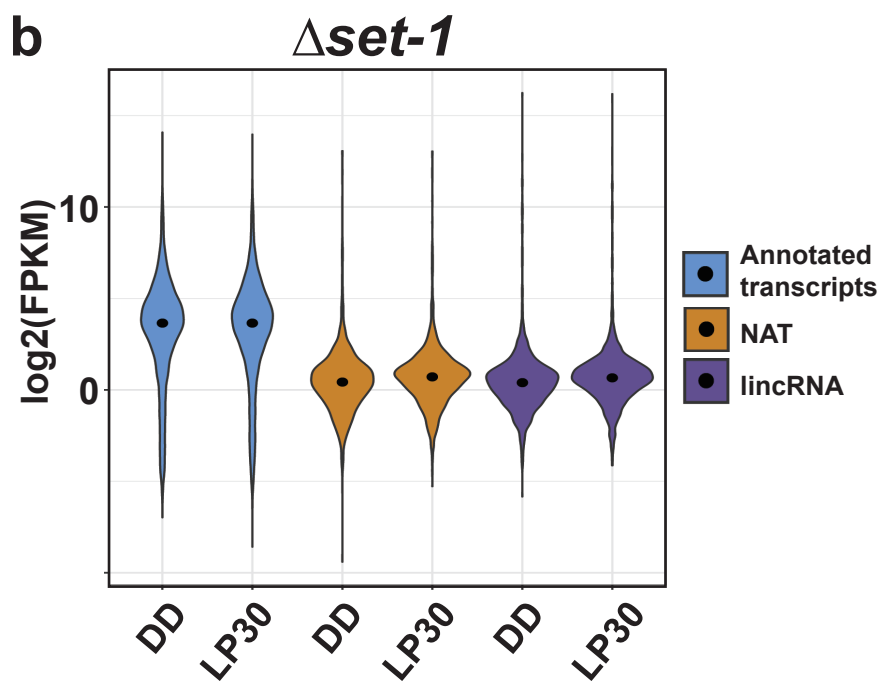

Supplement: Supplementary file 2 — Variation among transcript types in ∆kmt2/∆set-1 and ∆kmt1/∆dim-5. Violin plot depicting the expression levels (FPKM, log2) of transcripts belonging to existing annotated transcripts, NATs and lincRNAs in (a) kmt2/∆set-1 (b) kmt1/∆dim-5. (PDF 721 kb) [file 12864_2019_5729_MOESM2_ESM.pdf]

**a**

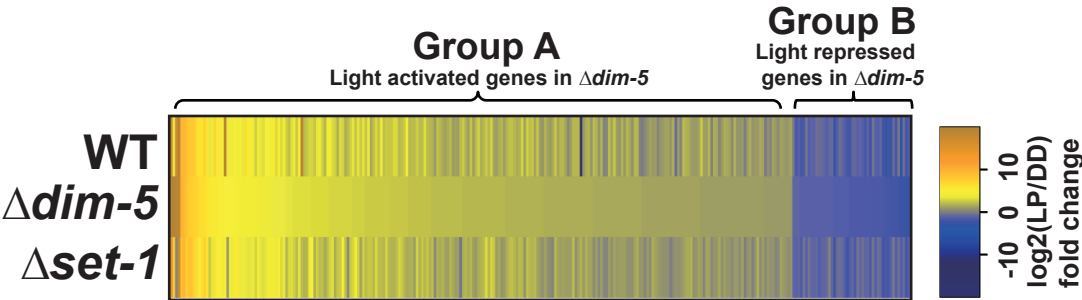

**b**

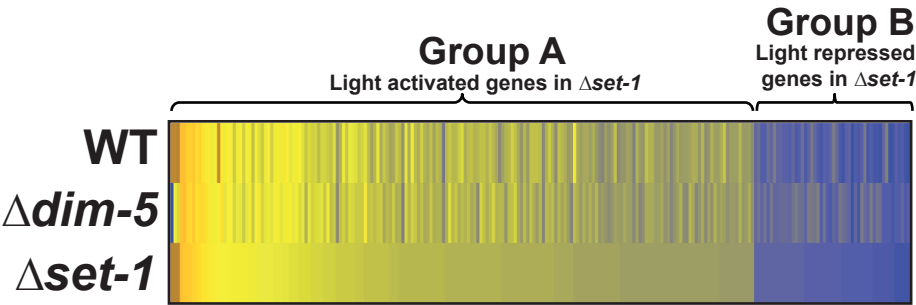

**c**

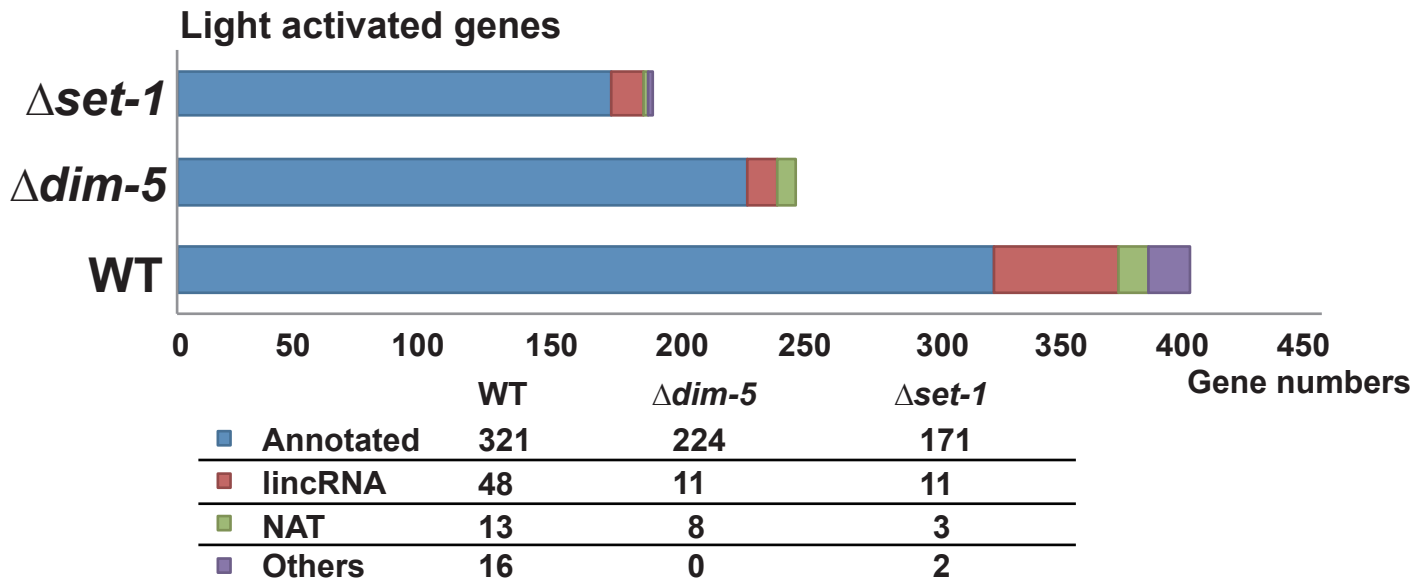

Supplement: Supplementary file 4 — Hierarchical clustering of light regulated genes in ∆kmt2/∆set-1 and ∆kmt1/∆dim-5. (a) Heatmap showing the clustering of genes that are differentially expressed in ∆kmt1/∆dim-5 DD versus LP30 and compared to same genes in WT and ∆kmt2/∆set-1. The expression levels are log2 fold change (q < 0.05). (b) Same as in A except clustering was done in ∆kmt2/∆set-1 DD versus LP30. Genes in Group A are light activated and Group B includes genes that are light repressed. Changes in expression between the two conditions are displayed with range [− 10,10], with levels above and below the mean shown in yellow or blue. (c) Bar plot depicting the number of different transcripts categorized as an existing annotated gene, lincRNA, NAT or other that were light activated in WT, ∆kmt1/∆dim-5 and ∆kmt2/∆set-1. The x-axis is the gene number and the y-axis is the strain. (PDF 483 kb) [file 12864_2019_5729_MOESM4_ESM.pdf]

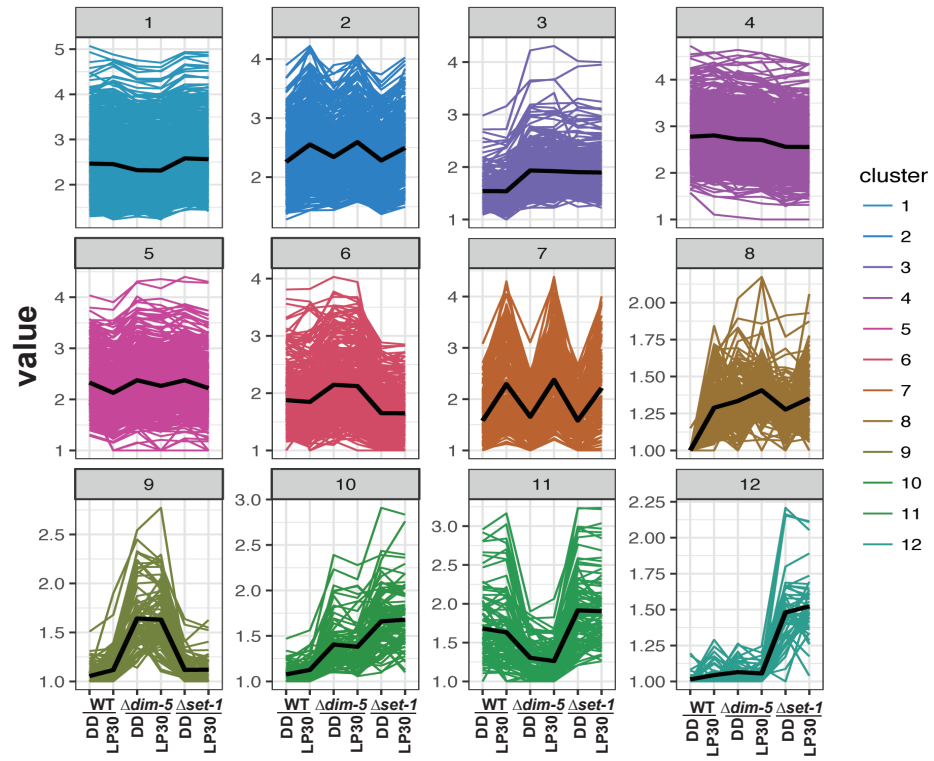

Supplement: Supplementary file 6 — Gene expression clusters of WT, ∆kmt1/∆dim-5 and ∆kmt2/∆set-1. Gene expression patterns were clustered into 12 groups based on the expression profile. The colored lines represent the pattern for each gene, and the black lines represent the median of all the genes in a given cluster. (PDF 1434 kb) [file 12864_2019_5729_MOESM6_ESM.pdf]

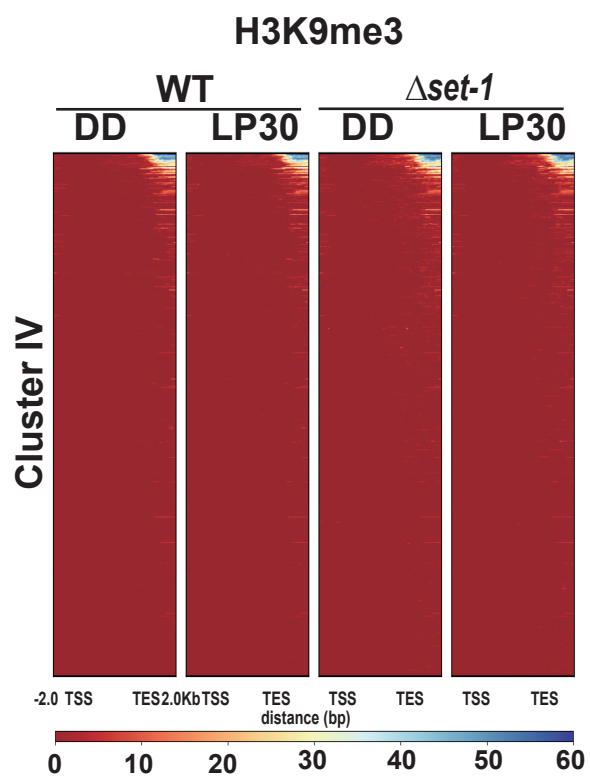

Supplement: Supplementary file 7 — The heatmaps display ChIP enrichment of H3K9me3 in WT and ∆kmt2/∆set-1. Regions included 2 kb upstream of TSS and downstream of TES. In this specific cluster, H3K9me3 is found downstream of the TES in a small subset of genes adjacent to constitutive heterochromatin. (PDF 665 kb) [file 12864_2019_5729_MOESM7_ESM.pdf]

**a**

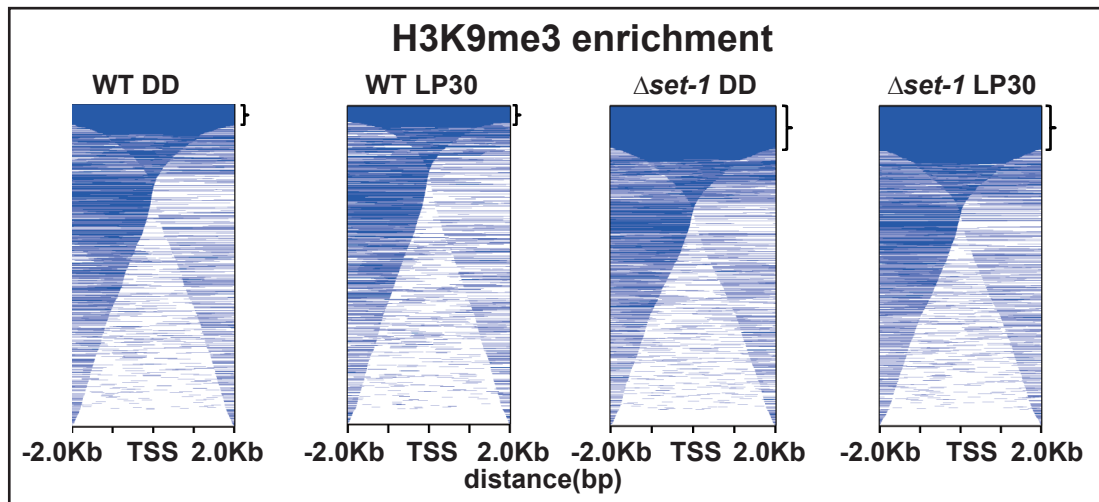

**b**

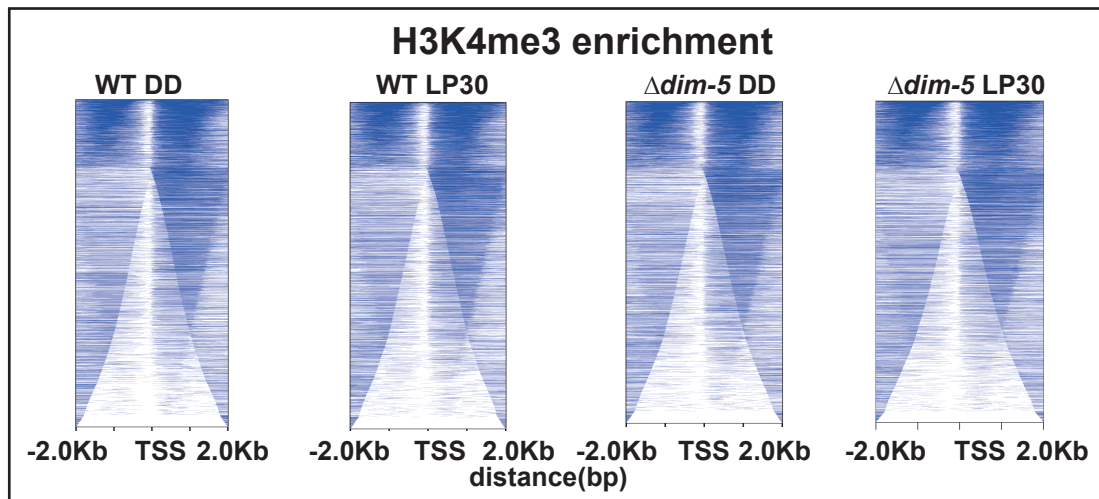

**c**

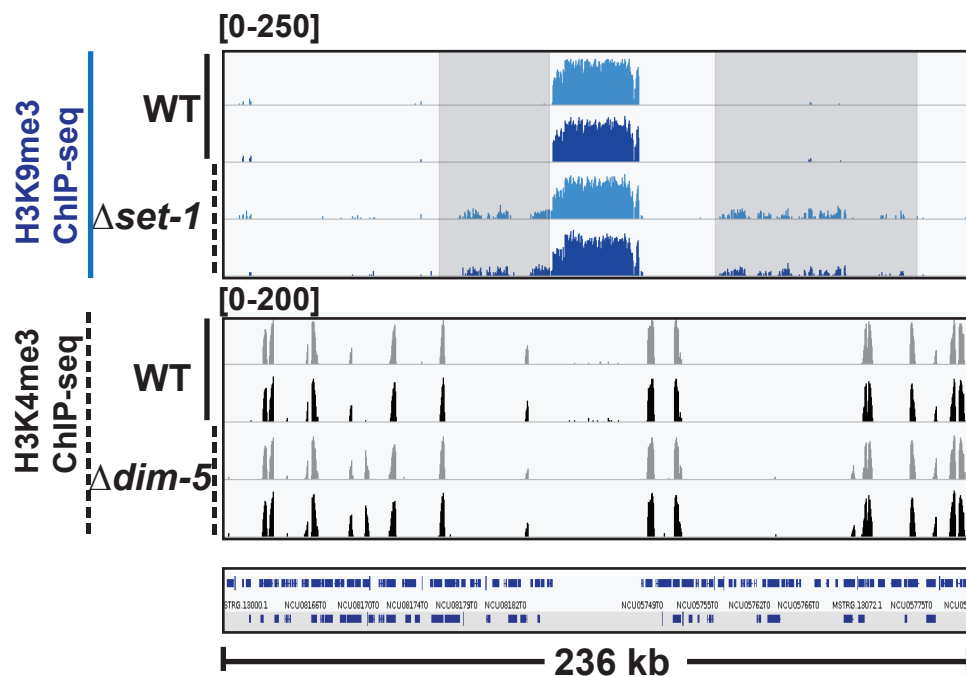

Supplement: Supplementary file 8 — H3K9me3 spreading in kmt2/∆set-1 strain. Heatmap display signal distribution for (A) H3K9me3 (B) H4K4me3 density plotted in a 2-kb windows centered on the TSS. The curly bracket(s) in panel A indicate the extent of H3K9me3 spreading in ∆kmt2/∆set-1. (C) Gene-level plot of a 236 kb region on chromosome VII (supercontig 12.7) showing H3K9me3 ChIP-seq (DD Blue and LP30 Navy) for the WT and ∆kmt2/∆set-1 and H3K4me3 ChIP-seq (DD Grey and LP30 black) for the WT and ∆kmt1/∆dim-5. The shaded boxes highlight representative examples of H3K9me3 spreading into euchromatic regions in ∆kmt2/∆set-1. (PDF 868 kb) [file 12864_2019_5729_MOESM8_ESM.pdf]

**a**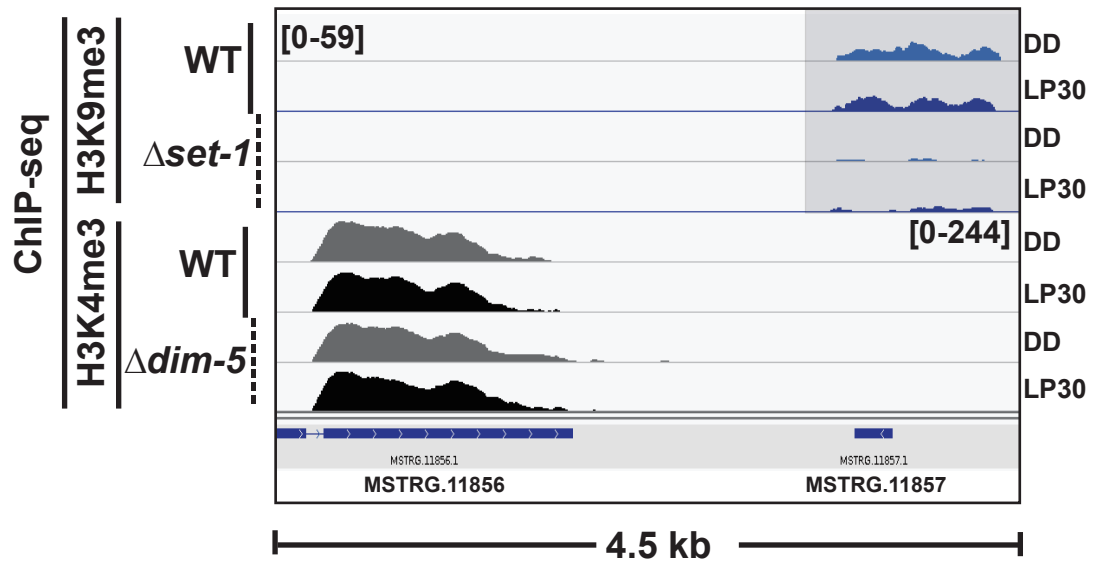**b**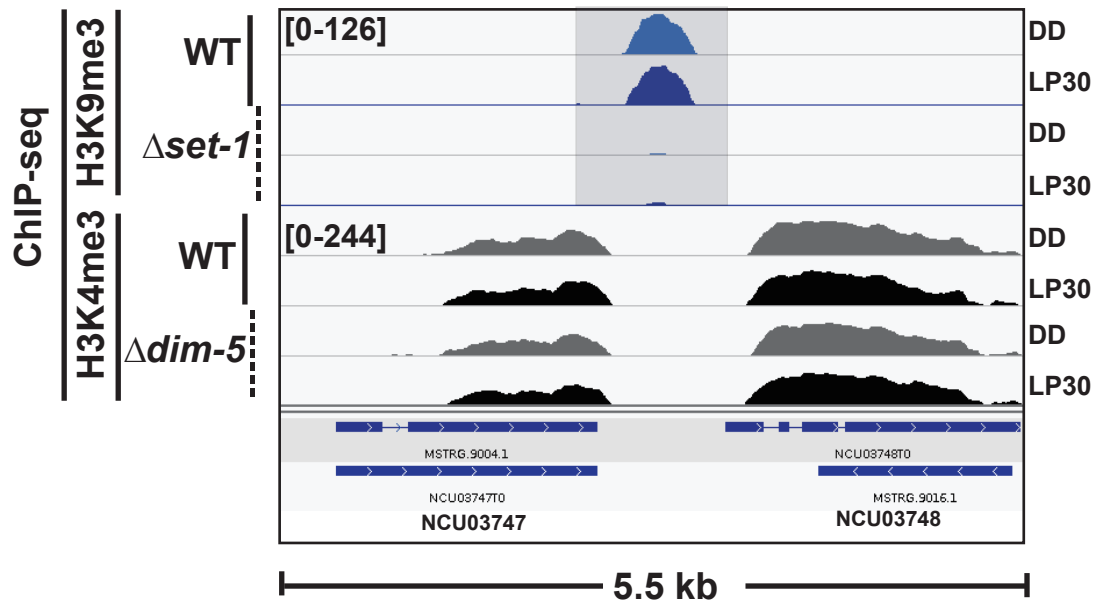**c**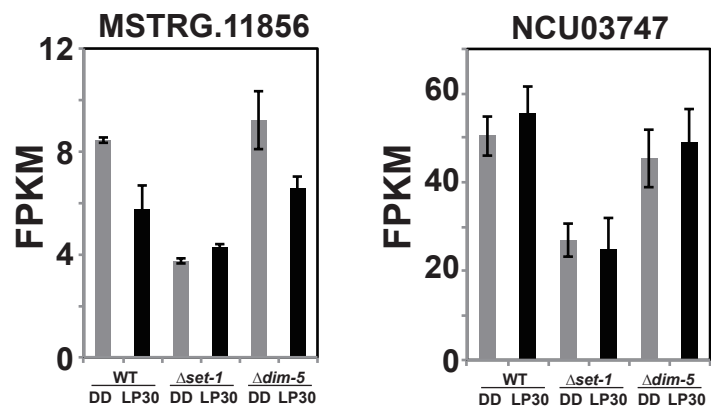

Supplement: Supplementary file 10 — Additional loci that have KMT2/SET-1-dependent heterochromatin. Gene-level diagram of H3K9me3 ChIP-seq (DD Blue and LP30 Navy) in WT and ∆kmt2/∆set-1, and H3K4me3 ChIP-seq (DD Grey and LP30 black) in WT and ∆kmt1/∆dim-5 for a presumptive lincRNA, (a) MSTRG.11857 and (b) NCU03747. It is clear from the traces that both have a significant decrease in H3K9me3 (p < 0.05) in ∆kmt2/∆set-1. (c) Expression bar plot of MSTRG.11856 and NCU03747, which have a decrease in expression when KMT2/SET-1-dependent heterochromatin is lost. (PDF 402 kb) [file 12864_2019_5729_MOESM10_ESM.pdf]

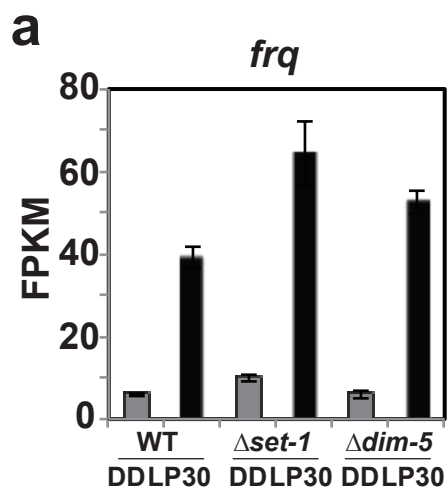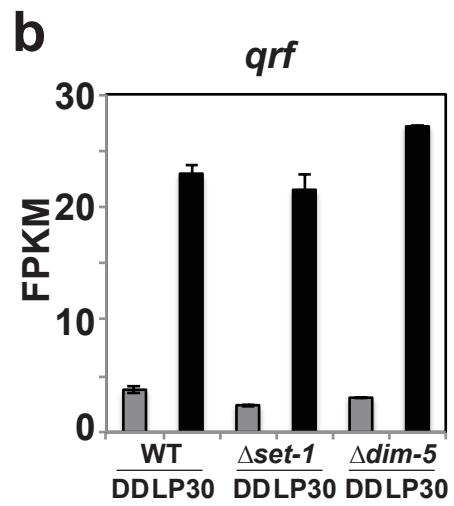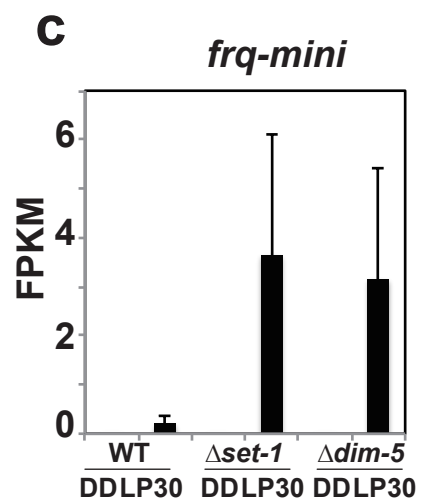

Supplement: Supplementary file 11 — Expression of transcripts arising from the frq locus. FPKM values for (a) frq, (b) qrf and (c) frq-mini are shown as bar plots in WT, kmt2/∆set-1 and kmt1/∆dim-5 for DD (grey) and LP30 (black). (PDF 388 kb) [file 12864_2019_5729_MOESM11_ESM.pdf]

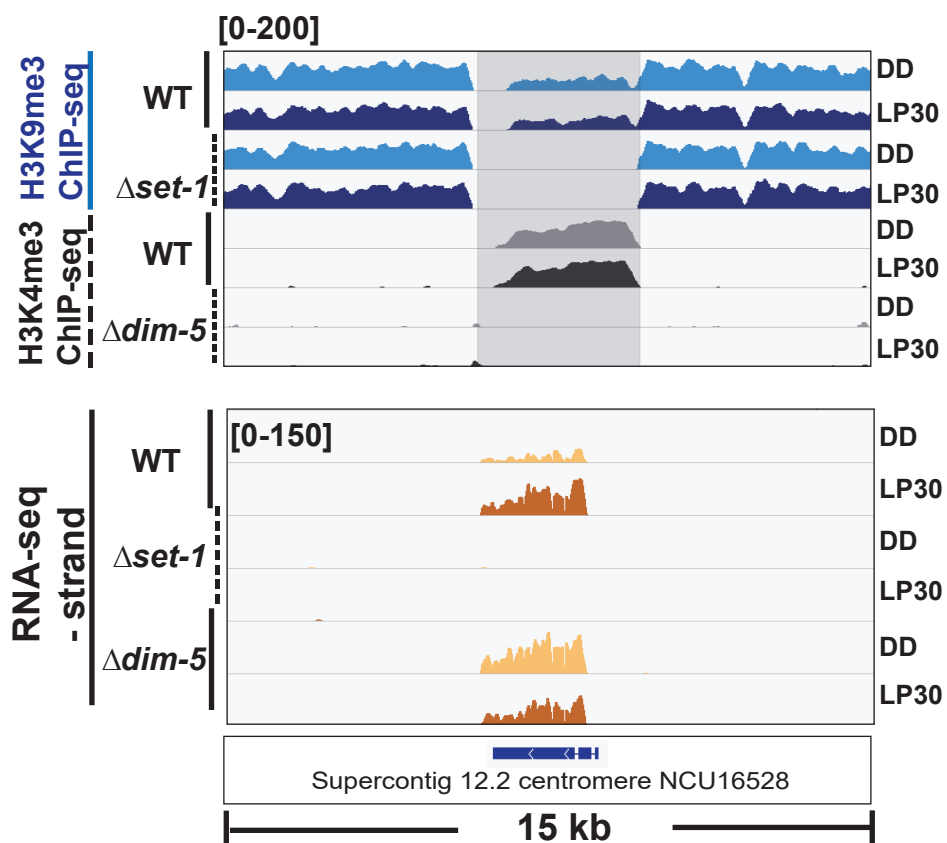

Supplement: Supplementary file 13 — Reciprocal dependence of H3K9me3 and H3K4me3. Gene level plot shows a centromeric gene on Chromosome II (supercontig 12.2). The IGV diagram displays H3K9me3 ChIP-seq (DD Blue and LP30 Navy) in WT and ∆kmt2/∆set-1 and H3K4me3 ChIP-seq (DD Grey and LP30 black) in WT and ∆kmt1/∆dim-5. The corresponding RNA-seq traces are also shown for NCU16528. (PDF 427 kb) [file 12864_2019_5729_MOESM13_ESM.pdf]

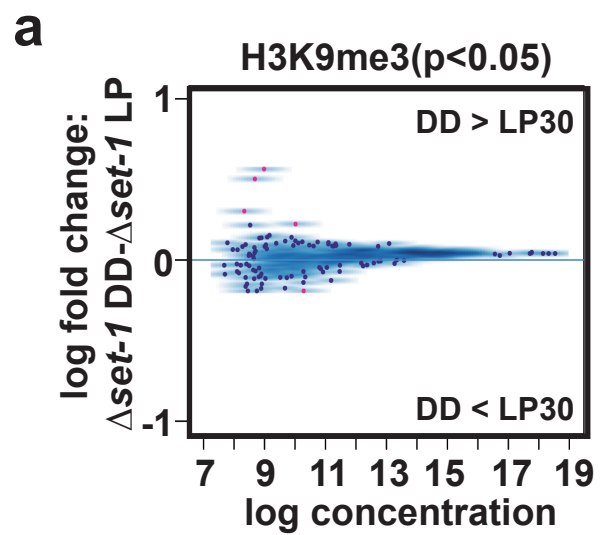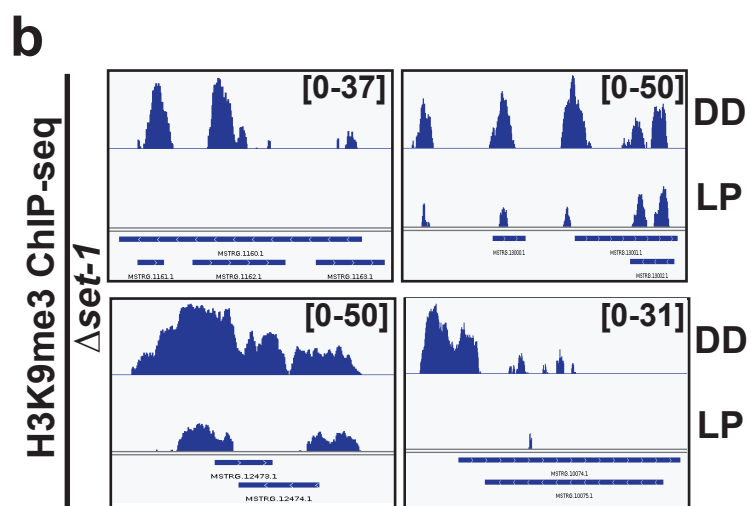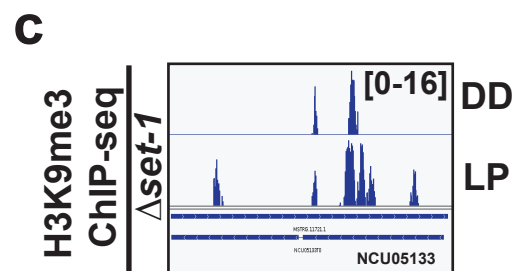

Supplement: Supplementary file 15 — Changes in H3K9me3 in response to light in ∆kmt2/∆set-1. (a) Quantitative difference in H3K9me3 levels from the ChIP-seq in ∆kmt2/∆set-1 upon light exposure. The MA plot shows log fold change (p < 0.05) in enrichment in DD (log fold change > 0) or LP (log fold change < 0). (b) IGV diagrams of 4 genes that had a decrease in H3K9me3 in ∆kmt2/∆set-1 in response to light (p < 0.05) (c) IGV diagram of NCU05133 which had an increase in H3K9me3 in ∆kmt2/∆set-1 (p < 0.05) in response to light. (PDF 543 kb) [file 12864_2019_5729_MOESM15_ESM.pdf]

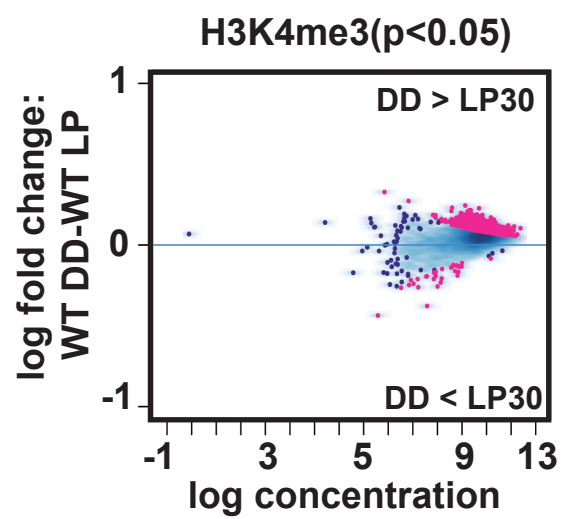

Supplement: Supplementary file 16 — Light induced changed in H3K4me3 in WT. Quantitative difference in H3K4me3 levels in WT DD versus WT LP30. The MA plot shows increase in H3K4me3 in DD (log fold change > 0) or in response to light (log fold change < 0). Spots shown in red have a p < 0.05. (PDF 449 kb) [file 12864_2019_5729_MOESM16_ESM.pdf]
